# Docking, Synthesis and Antiproliferative Activity of *N*-Acylhydrazone Derivatives Designed as Combretastatin A4 Analogues

**DOI:** 10.1371/journal.pone.0085380

**Published:** 2014-03-10

**Authors:** Daniel Nascimento do Amaral, Bruno C. Cavalcanti, Daniel P. Bezerra, Paulo Michel P. Ferreira, Rosane de Paula Castro, José Ricardo Sabino, Camila Maria Longo Machado, Roger Chammas, Claudia Pessoa, Carlos M. R. Sant'Anna, Eliezer J. Barreiro, Lídia Moreira Lima

**Affiliations:** 1 Instituto Nacional de Ciência e Tecnologia de Fármacos e Medicamentos (INCT-INOFAR). Universidade Federal do Rio de Janeiro, Laboratório de Avaliação e Síntese de Substâncias Bioativas (LASSBio) Rio de Janeiro, Brasil; 2 Programa de Pós-Graduação em Química, Instituto de Química, Universidade Federal do Rio de Janeiro, Rio de Janeiro, Brasil; 3 Departamento de Fisiologia e Farmacologia, Faculdade de Medicina, Universidade Federal do Ceará, Fortaleza, Brasil; 4 Departamento de Ciências Biológicas, Campus Senador Helvídio Nunes de Barros, Universidade Federal do Piauí, Picos, Brasil; 5 Instituto de Física, Universidade Federal de Goiás, Goiânia, Brazil; 6 Faculdade de Medicina, Departamento de Radiologia, Universidade de São Paulo, São Paulo, Brasil; 7 Departamento de Química, Universidade Federal Rural do Rio de Janeiro, Seropédica, Brasil; Univ of Bradford, United Kingdom

## Abstract

Cancer is the second most common cause of death in the USA. Among the known classes of anticancer agents, the microtubule-targeted antimitotic drugs are considered to be one of the most important. They are usually classified into microtubule-destabilizing (e.g., Vinca alkaloids) and microtubule-stabilizing (e.g., paclitaxel) agents. Combretastatin A4 (CA-4), which is a natural stilbene isolated from *Combretum caffrum*, is a microtubule-destabilizing agent that binds to the colchicine domain on β-tubulin and exhibits a lower toxicity profile than paclitaxel or the Vinca alkaloids. In this paper, we describe the docking study, synthesis, antiproliferative activity and selectivity index of the *N*-acylhydrazone derivatives (**5a–r**) designed as CA-4 analogues. The essential structural requirements for molecular recognition by the colchicine binding site of β-tubulin were recognized, and several compounds with moderate to high antiproliferative potency (IC_50_ values ≤18 µM and ≥4 nM) were identified. Among these active compounds, LASSBio-1586 (**5b**) emerged as a simple antitumor drug candidate, which is capable of inhibiting microtubule polymerization and possesses a broad *in vitro* and *in vivo* antiproliferative profile, as well as a better selectivity index than the prototype CA-4, indicating improved selective cytotoxicity toward cancer cells.

## Introduction

Microtubules (MTs) are cytoskeletal polymers formed by the polymerization of á- and β-tubulin heterodimers, which is followed by GTP hydrolysis; the polymerization occurs through two important steps: nucleation and elongation. MTs are found within all dividing eukaryotic cells, as well as in most differentiated cell types, and play crucial roles in cell division, cell motility, cellular transport, the maintenance of cell polarity, and cell signaling [1].

Microtubules are labile polymers that display two types of dynamic behaviors, which are called “treadmilling” and “dynamic” instability. The latter, is characterized by the alternating growing and shortening phases of the microtubule ends. The transition from a growing phase to a shortening phase is called a catastrophe, while a transition from a shortening phase to a growing phase is known as a rescue. Because microtubule dynamics play an important role in various cellular functions, such as mitosis, they are a potential target for development of anti-cancer drugs [1]–[4].

**Figure 1:**
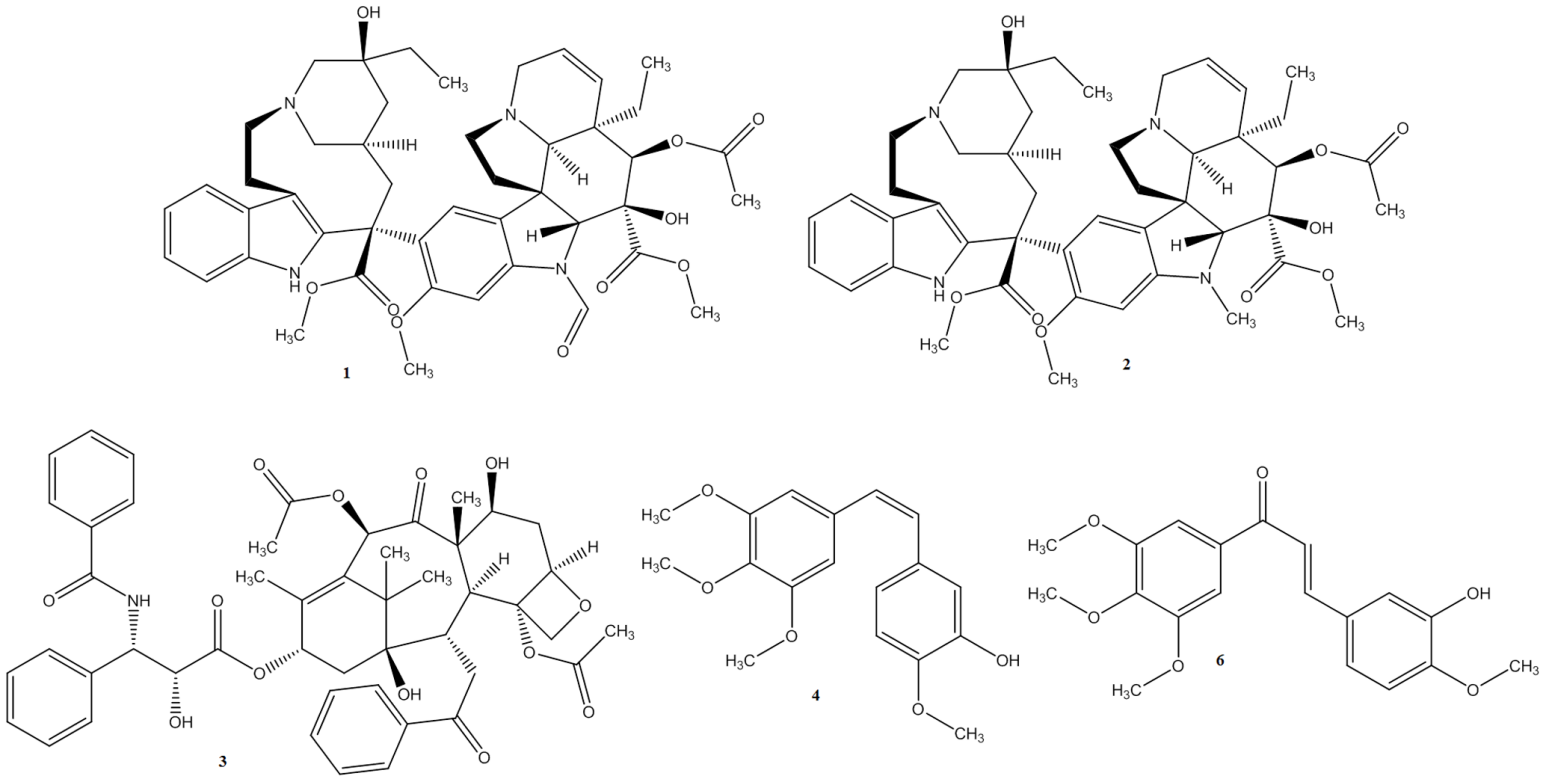
Anti-microtubule agents: vincristine (1), vinblastine (2), paclitaxel (3), CA-4(4) and its chalcone analogue (6).

Microtubule-targeting antimitotic drugs are usually classified into two main groups. One group, which is composed of microtubule-destabilizing agents, inhibits microtubule polymerization and includes compounds such as the Vinca alkaloids, vincristine (**1**) and vinblastine (**2**) (Figure 1); these two compounds were the first anti-microtubule agents approved to treat cancer. The second group encompasses the microtubule-stabilizing agents; these compounds stimulate microtubule polymerization and include paclitaxel, which is used to treat breast and ovarian cancer, non-small-cell lung cancer and Kaposi's sarcoma [4].

While vinblastine binds close to the exchangeable GTP site on the β-tubulin in a region called the Vinca-binding domain, paclitaxel (**3**, Figure 1) binds to the inner surface of the microtubules in a deep hydrophobic pocket on the βtubulin; this site is called the paclitaxel binding site [4]–[5].

During the development of orally bioavailable anti-microtubule agents that overcome the neurotoxicity and development of resistance commonly observed with the Vinca alkaloids, paclitaxel and their analogues, combretastatin A4 (CA-4, Figure 1) was discovered and is currently considered a promising lead-compound. This stilbene natural product, which was isolated from *Combretum caffrum*, binds to the colchicine domain on β-tubulin and exhibits a low toxicity profile [6]. Despite its potent antiproliferative activity, CA-4 (**4**) failed to exhibit anticancer efficacy in animal models because it has low water solubility, poor oral bioavailability, a short half-life and a double bond that isomerizes (*Z* to *E*) in vivo; this isomerization causes a loss of affinity for β-tubulin and consequently a loss of cytotoxic activity [7]–[11].

This paper describes the docking studies, synthesis and assessment of antiproliferative activity and selectivity index of *N*-acylhydrazone derivatives (**5a–r**) designed as CA-4 analogues.

**Figure 2:**
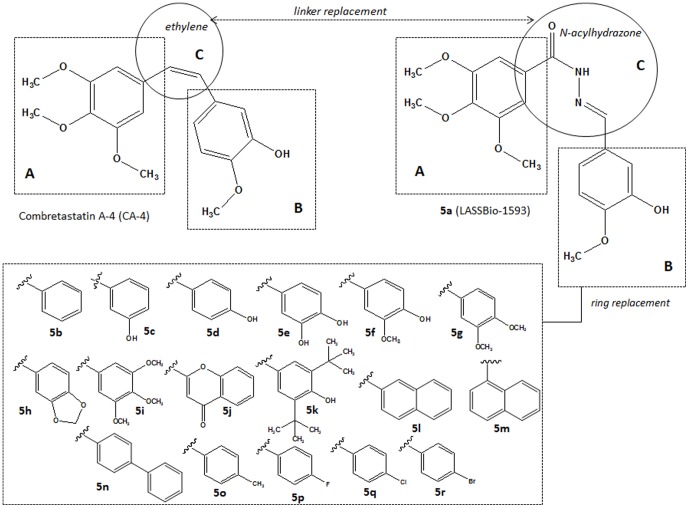
Initial conception and molecular design of *N*-acylhydrazone derivatives 5a–s.

The initial design conception of the *N*-acylhydrazone derivatives (**5a–r**) is depicted in Figure 2. The most important structural modification was the replacement of ethylene linker between the aromatic subunits **A** and **B** with a more stable *N*-acylhydrazone (NAH) scaffold, generating compound **5a**. To design a congeneric series (**5b–r**), several modifications were introduced in the substitution of aromatic subunit **B** based on docking studies with the colchicine binding site of the β-tubulin protein.

## Results and Discussion

**Figure 3:**
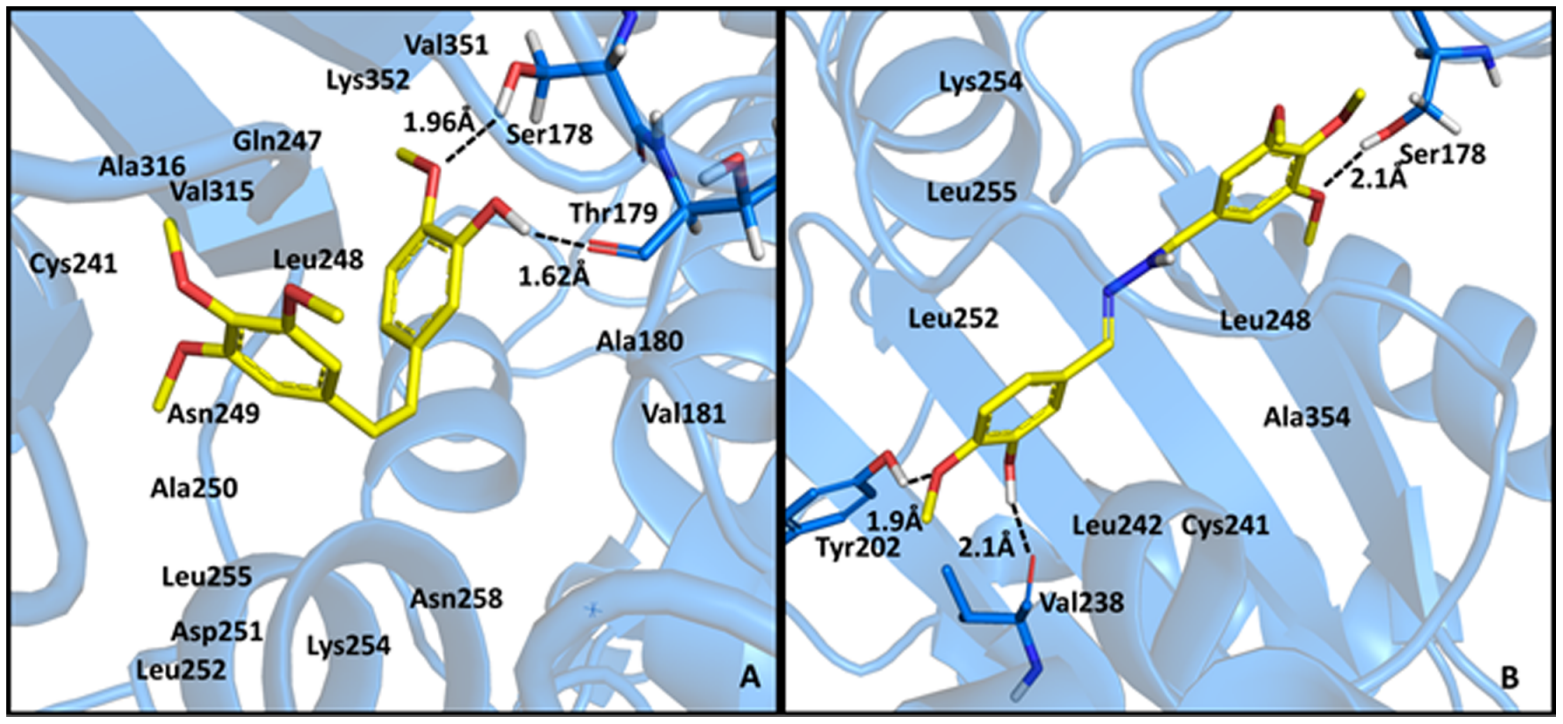
Polar interactions between CA-4 (A) or LASSBio-1593 (B) with the colchicine binding site of β-tubulin (PDB code: 1sa0).

All designed compounds were predicted to favorably interact with the DAMA-colchicine binding site in β-tubulin (PDB code: 1sa0) [12]. In the best-ranked solutions, there were few polar interactions, and the complementarity between the ligand and the receptor protein involved extensive, nonspecific interactions with hydrophobic groups. These results were in accordance with the DAMA-colchicine interaction mode observed in the co-crystallized structure: there is only one polar interaction, which occurred between the Cys241 SH group and one of the methoxy groups on the ligand (data not shown). Previously, the proximity between these groups was explored to establish a cross-link between the colchicine derivatives substituted at this methoxy position and Cys241 [13]. Combretastatin A4 (CA-4) was also predicted to interact primarily with the hydrophobic groups; its trimethoxy ring (ring **A**) occupied a similar position to the corresponding colchicine ring, and its second ring (ring **B**) formed two hydrogen bonds, which were between its phenolic hydroxyl group and Thr179 peptide carbonyl group, as well as between the adjacent methoxy group and Ser178 side chain [7], [10], [11]. Similar studies performed with the *E*-isomer CA4 show the loss of interactions with residues Ser178 and Thr179, which may somehow explain the inactivity of this isomer (see Figure S2 in supporting material). Additionally, its *N*-acylhydrazone analogue, which was LASSBio-1593 (**5a**), interacted with Ser178 through a methoxy group on ring **A**, and its isovaline ring (ring **B**) formed two hydrogen bonds, one with Val238 and the other with Tyr202 (Figure 3).

Based on the docking studies with compound **5a**, several modifications were enacted on the 4-methoxy-3-hydroxy-phenyl moiety (ring **B**, Figure 2) to vary the oxygenated pattern (**5c–j**) and explore more lipophilic substituents (**5b**, **5l–r**), while making allowances for the hydrophobic nature of colchicine binding pocket (Table 1). The modification of the linker between rings **A** (*i.e.*, 3,4,5-trimethoxyphenyl) and **B** (*i.e.*, 3-hydroxy,4-methoxyphenyl) resulted in the introduction of an *N*-acylhydrazone (NAH) subunit to replace the ethylene bridge (CH = CH). As expected, this type of modification caused significant conformational changes and altered the spatial arrangement of rings **A** and **B** during molecular recognition by β-tubulin (Figure 2). These findings are supported by data from the literature describing the anti-tubulin activity of *E*-chalcones (e.g., **6**, Figure 1) [14]–[16]. Additionally, the introduction of halogens substituents at position 4 of the **B** ring took advantage of the metabolic protection that might be exerted by these substituents, preventing aromatic hydroxylation at C4 catalyzed by the CYP450 enzymatic complex [17].

**Table 1:** Scores estimated by molecular docking (ChemScore fitness function) for colchicine binding site of β-tubulin, cLogP, cLogD_7.0_, molar refractivity and the aqueous solubility of CA-4 and its *N*-acylhydrazone analogues **5a–r**.

Compounds	Score^1^ (S.D.)	cLogP^2^	cLogD^2^	MR (S.D.)^3^	Aq. Solubility (mg/mL)^4^
**5a**	24.64 (0.76)	2.76	2.29	93.03 (0.5)	2.84×10^−2^
**5b**	24.54 (1.10)	3.15	2.78	86.36 (0.5)	3.95×10^−2^
**5c**	24.43 (0.33)	2.73	2.62	87.21 (0.5)	5.68×10^−3^
**5d**	35.26 (0.94)	2.72	2.64	87.21(0.5)	1.5×10^−2^
**5e**	25.29 (0.44)	2.56	2.51	88.07 (0.5)	3.94×10^−3^
**5f**	24.47 (0.53)	2.76	2.71	93.03 (0.5)	1.35×10^−3^
**5g**	23.35 (0.63)	2.95	2.17	97.99 (0.5)	1.94×10^−3^
**5h**	22.76 (0.36)	3.02	2.24	91.2 (0.5)	1.34×10^−3^
**5i**	22.54 (0.64)	2.82	2.23	103.8 (0.5)	8.94×10^−5^
**5j**	24.21 (0.57)	6.11	5.55	99.88 (0.5)	7.83×10^−4^
**5k**	27.96 (0.84)	4.37	3.04	124.04 (0.5)	2.36×10^−5^
**5l**	29.19 (0.35)	4.33	3.96	102.25 (0.5)	4.89×10^−4^
**5m**	29.21 (0.91)	4.37	3.86	102.25 (0.5)	6.90×10^−5^
**5n**	30.66 (1.29)	5.00	4.50	111.47 (0.5)	6.06×10^−5^
**5o**	26.23 (0.99)	3.60	3.05	90.79 (0.5)	6.93×10^−3^
**5p**	22.98 (1.21)	3.34	3.02	86.23 (0.5)	2.09×10^−2^
**5q**	24.90 (0.73)	3.89	3.69	90.96 (0.5)	2.84×10^−2^
**5r**	25.08 (0.66)	4.11	3.69	93.92 (0.5)	2.62×10^−4^
**CA-4**	26.45 (1.53)	3.47	2.50	92.24 (0.3)	5.44×10^−3^

1 Values shown are the mean of 5 runs;

2 cLogP and cLogD (pH 7.0) were calculated using MetaSite Program (license number: URJ181011);

3 molar refractivity (MR) calculated with ChemSketch 12.0 (Freeware Version);

4 Solubility was determined by ultraviolet spectroscopy, as described by Schneider and co-workers.

To identify the most energetically favorable pose (*i.e.*, pose prediction), each pose of the *N*-acylhydrazone derivatives **5a–r** within the colchicine binding site of β-tubulin was evaluated (*i.e.*, scored) based on their complementarity to the target with respect to their shape and properties, such as electrostatics. It is noteworthy that score is the most adequate way of selecting the best pose, since the scores are assigned according to the interaction mode of a ligand with the binding site, as measured by fitness function. The fitness function was selected after redocking experiments with colchicine in the binding site of β-tubulin (PDB code: 1sa0). The RMSD between the experimental structure and the top scored pose, determined after redocking experiments with the four fitness functions available in GOLD 5.0.1 program (*i.e.* Chemscore, Goldscore, ASP and ChemPLP), revealed that Chemscore was the fitness function with the best performance in this study (RMSD = 1.0606).

Giving a good score to a compound indicates that it exhibited good binding with the protein, and the results were compared to the data obtained with CA4 (Table 1). As depicted in Table 1, five compounds (**5d**, **5k**, **5l**, **5m** and **5n**) were predicted to display better binding than CA4. In this group, the most favorable complementary interaction was observed with compound **5d** (LASSBio-1588), which forms a hydrogen bond between the hydroxyl group on ring B (*i.e.*, 4-hydroxyphenyl) with Val 662. However, compounds **5k**, **5l**, **5m** and **5n** display complementary interactions using the lipophilic nature of ring B to exploit the hydrophobic pocket composed by residues Leu242, Val238, and Leu255 at the colchicine site of β-tubulin (data not shown). These data agree with the work of Dorléans and coworkers: ligands of the colchicine binding site establish few polar interactions within the protein-ligand complex, and van der Waals interactions are more relevant during molecular recognition [18]. The worst scores were observed with compounds **5g**, **5h**, **5i** and **5p**, which possessed polar groups on ring **B** that could not act as hydrogen bond donors. The score values determined during the docking studies and some physicochemical properties (cLogP, cLogD, MR and the aqueous solubility) for compounds **5a–r** are summarized in Table 1 (see also Figure S3 in supporting material).

**Figure 4:**
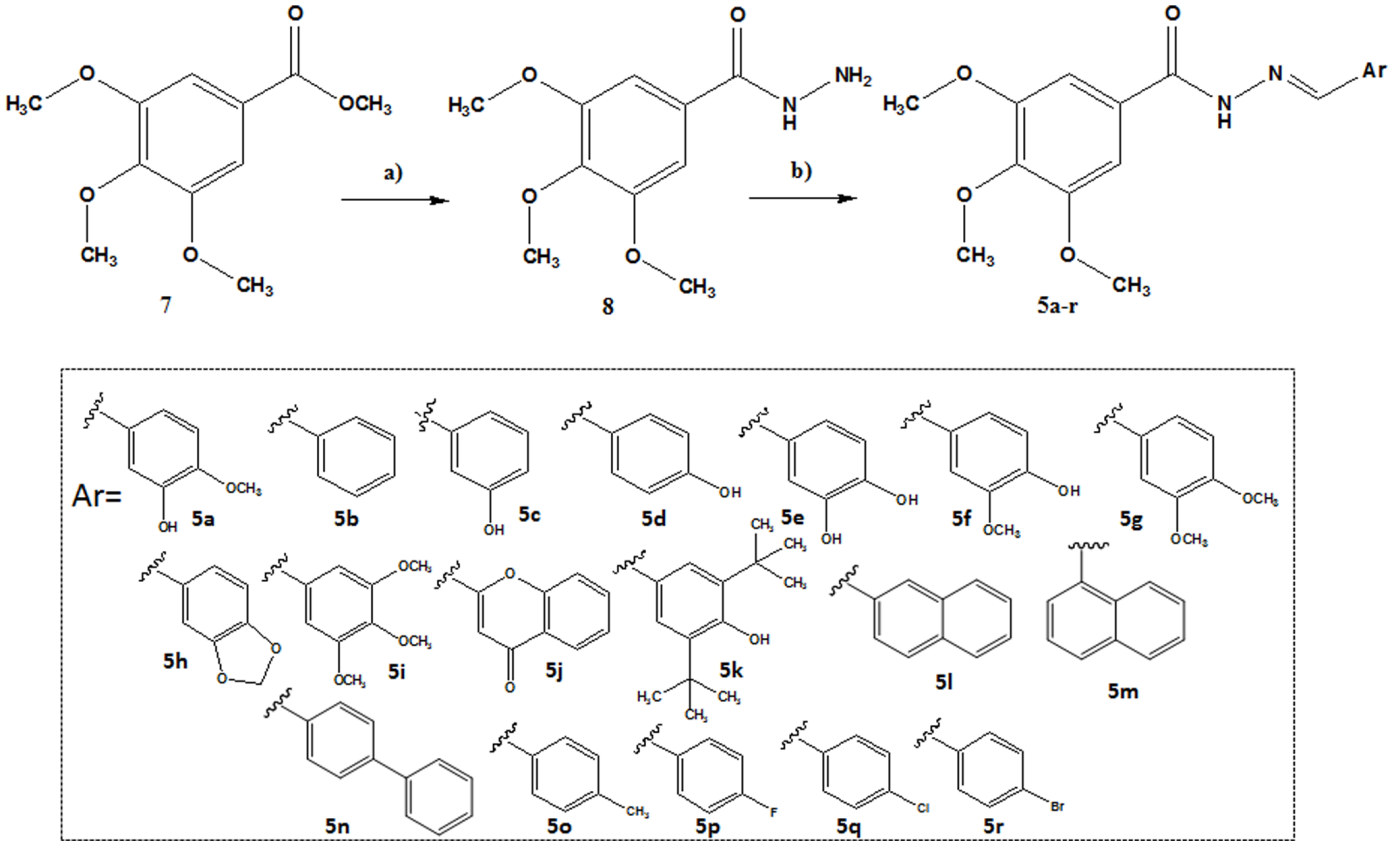
Conditions and reagents: a) 80% aq. N_2_H_4_.H_2_O, EtOH, reflux, 2 h, 93%. **b) ArCHO, EtOH, HCl (cat), r.t., 0.5–4 h, 62–95%**.

The *N*-acylhydrazones (**5a–r**) were obtained at a two-step linear route (Figure 4) [19], using methyl 3,4,5-trimethoxybenzoate ester (**7**) as the starting material. While exploring a hydrazinolysis reaction, ester **7** was refluxed with hydrazine hydrate 80% in ethanol, providing the 3,4,5-trimetoxybenzohydrazide (**8**) in 93% yield. The hydrazide (**8**) was condensed with the appropriate aldehydes, which were selected in accordance with the molecular design depicted in Figure 1, in the presence of ethanol and catalytic hydrochloric acid to furnish the CA-4 analogues **5a–r** in high yields.

Compounds **5a–r** were characterized by ^1^H NMR, ^13^C NMR and IR spectroscopy and their purity was determined by HPLC, with a reverse-phase column at different systems of mobile phase. All *N*-acylhydrazone derivatives (**5a–r**) were obtained as a single diastereoisomer (*Z* or *E*), as indicated by the analysis of the ^1^H and ^13^C NMR spectra; no duplicate signals attributed to the hydrogen or carbon atom of the imine (N = CH) were observed. The stereochemistry of the imine double bond was subsequently assigned based on our previous results [23] and the X-ray crystallographic studies performed with **5b** (LASSBio-1586).

**Figure 5:**
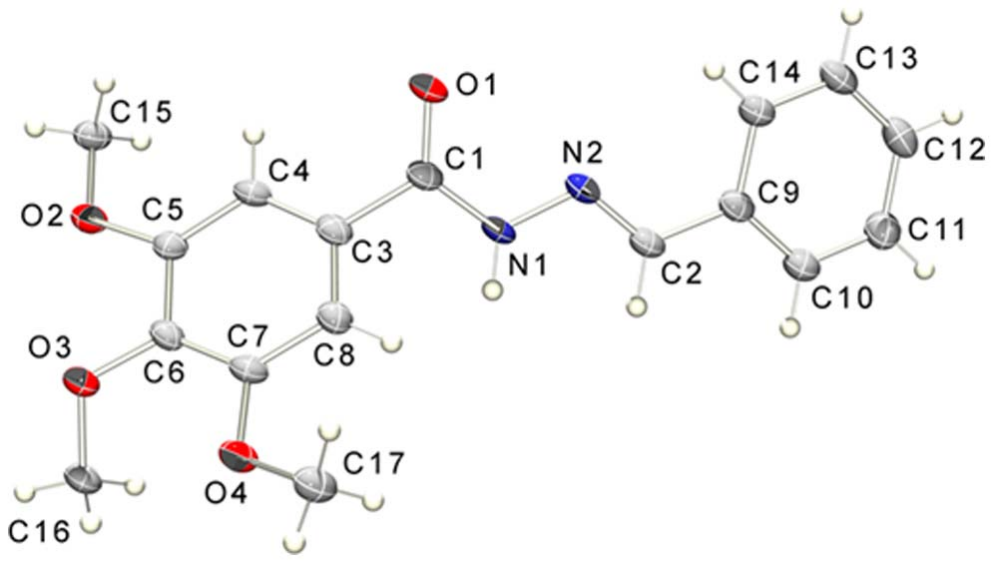
ORTEP view of compound 5b with the atom displacement ellipsoids drawn at a 50% probability level.

A single crystal of compound **5b** (LASSBio-1586) was obtained and subjected to X-ray diffraction; the ORTEP [20], [37] view is shown in Figure 5. Crystallographic analysis confirmed that the configuration about the C2 = N2 double bond [distance 1.273(3) Å] was *E* and revealed a nearly flat conformation of the benzoylhydrazide moiety, which was described by the least squares plane through the atoms O1/N1/C1/N2/C2/C9/C10/C11/C12/C13/C14 with a r.m.s. deviation of 0.066 Å, as well as C2—C9 bond torsion angles of 3.4 (3)° and −174.9 (2)°, respectively. The trimethoxyphenyl ring was rotated outward from this plane by 45.31(6)°, reducing the π-orbital contribution to this bond and allowing an elongation of the C1—C3 bond [1.499 (3) Å] relative to the expected bond length. One feature of acentric crystal structures occurs in this case, which is that torsional angles of the methoxy groups are unique, as given by: C4—C5—O2—C15 of 5.1 (3)°, C7—C6—O3—C16 of −56.0 (3)° and C8—C7—O4—C17 of 13.9 (3)°. The molecules are connected through an N—H…O intermolecular hydrogen bond with the carbonyl group and are arranged in a linear array though crystal axis a. The parallel arrays are bound by weak van der Waals interactions between methyl group C15 and the O2 oxygen atom from a neighboring molecule, demonstrating the availability of this group for intermolecular interactions once the methoxy group in the *para* position is rotated to the opposite side. Crystallographic data of compound **5b** (excluding structure factors) can be seen in supporting information. Crystallographic data of compound **5b** (excluding structure factors) can be seen in supporting information.

The antiproliferative activity of compounds **5a–r** was determined based on an MTT assay [21] and using CA-4 as standard against the tumor cell lines: HL-60 (human leukemia), SF-295 (human glioblastoma), MDA-MB435 (melanoma), PC3M (prostate cancer), OVCAR-8 (ovaries adenocarcinoma), NCI-H258M (pulmonary bronchio-alveolar carcinoma) and HCT-8 (adenocarcinoma ileocecal) (Table 2). To determine the selectivity index of compounds **5a–r**, their antiproliferative profile was also evaluated toward human lymphocytes (Table 2).

**Table 2:** *In vitro* antiproliferative potency (IC_50_-µM) of compounds **5a–r** and the standard CA-4 against tumor cell lines and human lymphocytes.

Compounds	*HL-60*	*SF295*	*HCT-8*	*MDA-MB435*	*PC3M*	*OVCAR-8*	*NCI-H358M*	*Lymphocytes*
**5a**	4.72	1.55	2.08	0.39	2.22	1.44	1.58	2.58
**5b**	0.29	0.26	0.45	0.064	0.8	0.29	0.35	1.34
**5c**	1.63	13.05	4.3	0.12	7.51	5.78	9.5	4.48
**5d**	2.63	15.95	6.54	0.88	4.57	6.18	11.75	13.38
**5e**	9.3	>25	>25	11.78	24.4	>25	>25	7.36
**5f**	9.85	13.57	9.27	6.52	16.96	14.6	11.85	36.51
**5g**	4.43	18.08	7.05	2.11	12.55	7.11	10.18	17.98
**5h**	3.07	0.86	55.81	0.11	1.14	1.09	2.15	1.31
**5i**	>25	>25	>25	>25	>25	>25	>25	>61.82
**5j**	>25	>25	23.35	>25	>25	>25	>25	>65.38
**5k**	>25	>25	>25	>25	>25	>25	>25	>56.49
**5l**	0.015	0.057	0.011	0.004	0.008	0.0054	0.079	0.010
**5m**	0.018	0.085	0.050	0.043	0.027	0.026	0.63	0.010
**5n**	>25	>25	>25	>25	>25	>25	>25	>64.07
**5o**	0.0048	0.093	0.046	0.035	0.0127	0.0082	0.891	0.0073
**5p**	1.27	2.69	2.02	1.58	4.48	0.96	2.16	3.82
**5q**	0.036	0.072	0.046	0.018	0.0275	0.024	1.055	0.060
**5r**	0.0109	0.059	0.022	0.0183	0.0127	0.0073	0.167	0.0314
**CA-4**	0.0021	0.0062	0.0053	0.0079	0.0047	0.00037	0.008	0.0032

As shown in Table 2, all compounds except for derivatives **5i**, **5j**, **5k** and **5n** exhibited moderate to high antiproliferative potency with IC_50_ values ≤18 µM and ≥4 nM. These results are in agreement with Jin and co-workers [22], who described the antiproliferative activity of some NAH containing the trimethoxyphenyl subunit against PC3, A431 and BGC823 tumor cells for the first time. The *N*-acylhydrazones with hydrophobic substituents on ring B (*i.e.*, **5l**, **5m**, **5o**, **5p**, **5q** and **5r**) were more potent, which was predicted by the score values obtained from the docking studies. The *in silico* study failed to predict the cytotoxic activity of compound **5n** and **5d**, which scored as better binders than CA-4. The inactivity of compound **5n** (IC_50_>25 µM) suggested that there were steric constraints in the recognition between the ligand and the active site of β-tubulin because compounds with bulkier groups (for MR values see Table 1) attached to the imine (*i.e.* **5i**, **5j, 5k** and **5n**) displayed the worst activities. Moreover, compounds **5i**, **5k** and **5n** bind differently from CA4 and **5b** at the colchicine binding site, with no interaction with residues Ser178 and/or Thr 179 (see Figure S4 in supporting material). The addition of a 2-chromone subunit caused the loss of antiproliferative potency, while the inclusion of oxygenated substituents at the phenyl ring (ring B; **5a**, **5c**, **5d**, **5e**, **5f**, **5g**, **5h**) did not significantly interfere with the cytotoxic potency compared to compound **5b**; however, these compounds were still significantly less active than CA-4 (Table 2).

To investigate the selective cytotoxic activity of the *N*-acylhydrazones derivatives (**5a–r**), their antiproliferative potency was also assessed toward human lymphocytes and the results were compared to the data from CA-4 (Table 3). The selectivity index (SI), which was the IC_50_ for human lymphocytes/IC_50_ for cancer cell lines after treatment with CA-4 and *N*-acylhydrazones (**5a–r**), was calculated, as depicted in Table 3. Excluding the compounds that were inactive or slightly cytotoxic (**5i**, **5j**, **5k** and **5n**), the more lipophilic (cLogP ≥3.15 ≤4.37, Table 1) compounds (**5b**, **5l**, **5m**, **5o**, **5p**, **5q**, **5r**) exhibited cytotoxic potency against human lymphocytes similar to the lead compound, CA-4. Notably, CA-4 was proven to be a non-selective cytotoxic agent, with higher antiproliferative potency against human lymphocytes versus tumor cell lines, except for HL-60 and OVCAR-8 (Table 3). In contrast, LASSBio-1586 (**5b**) exhibited a cytotoxic selectivity index from 2.4 to 42 times greater than CA-4 (Table 3; SI values for **5e** versus SI values for CA-4). The best comparative selectivity indices (CA-4 vs **5b**) were obtained from the SF-295 (SI = 13), MDA-MB435 (SI = 42) and NCI-H258M (SI = 9.5) tumor cell lines, and the worst results were found for OVCAR-8 (SI = 0.5).

**Table 3:** The selectivity index (SI) of CA-4 and *N*-acylhydrazones (**5a–r**).

Compounds	SI	SI	SI	SI	SI	SI	SI
	Lymphocyte/	Lymphocyte/	Lymphocyte/	Lymphocyte/	Lymphocyte/	Lymphocyte/	Lymphocyte/
	HL-60	SF-295	HCT-8	MDA-MB345	PC3M	OVCAR-8	NCI-H358M
CA-4	1.44±0.05	0.44±0.04	0.59±0.01	0.42±0.02	0.72 v 0.02	0.030±0.70	0.52±0.12
**5a**	0.56±0.01	1.89±0.19	1.31±0.11	6.30±0.30	1.40±0.30	1.65±0.15	1.70±0.10
**5b**	4.11±0.39	4.88±0.42	2.86±0.13	20.07±0.93	1.60±0.10	4.39±0.21	4.05±0.25
**5c**	2.60±0.10	0.32±0.02	0.96±0.35	32.50±4.50	0.58±0.01	0.80±0.005	0.48±0.01
**5d**	5.15±0.05	0.82±0.02	2.07±0.07	16.06±0.86	2.96±0.86	2.22±0.02	1.16±0.06
**5e**	0.82±0.02	0.02±0.00	0.02±0.00	0.06±0.00	0.30±0.005	0.2±0.00	0.30±0.00
**5f**	3.77±0.07	2.75±0.05	3.91±0.10	5.52±0.08	2.25±0.05	2.53±0.03	3.12±0.02
**5g**	4.10±0.10	0.99±0.005	2.50±0.005	8.41±0.09	1.44±0.04	2.55±0.05	1.80±0.005
**5h**	0.43±0.01	1.47±0.02	0.02±0.002	13.17±1.37	1.14±0.06	1.16±0.04	0.62±0.02
**5i**	1	1	1	1	1	1	1
**5j**	1	1	2.8	1	1	1	1
**5k**	1.1	1	1	1	1	1	1
**5l**	0.61±0.09	0.18±0.02	0.80±0.09	1.96±0.53	1.15±0.15	1.95±0.05	0.11±0.01
**5m**	0.52±0.07	0.11±0.01	0.21±0.01	0.26±0.06	0.37±0.03	0.42±0.02	0.01±0.005
**5n**	1.0	1.0	1.0	1.0	1	1	1
**5o**	1.59±0.09	0.08±0.00	0.15±0.005	0.21±0.01	0.56±0.04	0.91±0.01	0.009±0.001
**5p**	3.11±0.11	1.46±0.06	1.85±0.04	2.34±0.06	0.89±0.01	4.38±0.38	1.76±0.04
**5q**	1.85±0.15	0.85±0.05	1.25±0.05	3.07±0.22	2.10±0.10	2.75±0.25	0.05±0.005
**5r**	3.07±0.83	0.49±0.05	1.57±0.17	1.81 0.11	2.29±0.20	4.02±0.28	0.19±0.01

Considering the IC_50_ (≤0.8 µM and ≥0.064 µM, Table 2) and the SI values (Table 3), LASSBio-1586 (**5b**) was selected as the most promising compound, and its ability to inhibit tubulin polymerization was investigated. The tubulin polymerization assay was performed by CEREP® employing a single concentration of **5b** (C = 30 µM), using vinblastine as positive control. In this assay, LASSBio-1586 (**5b**) inhibited 91% of the tubulin polymerization, validating the rational design employed in the molecular design of the derivatives **5a–r** (data not shown; available in the supplementary information, Figure S1).

**Figure 6:**
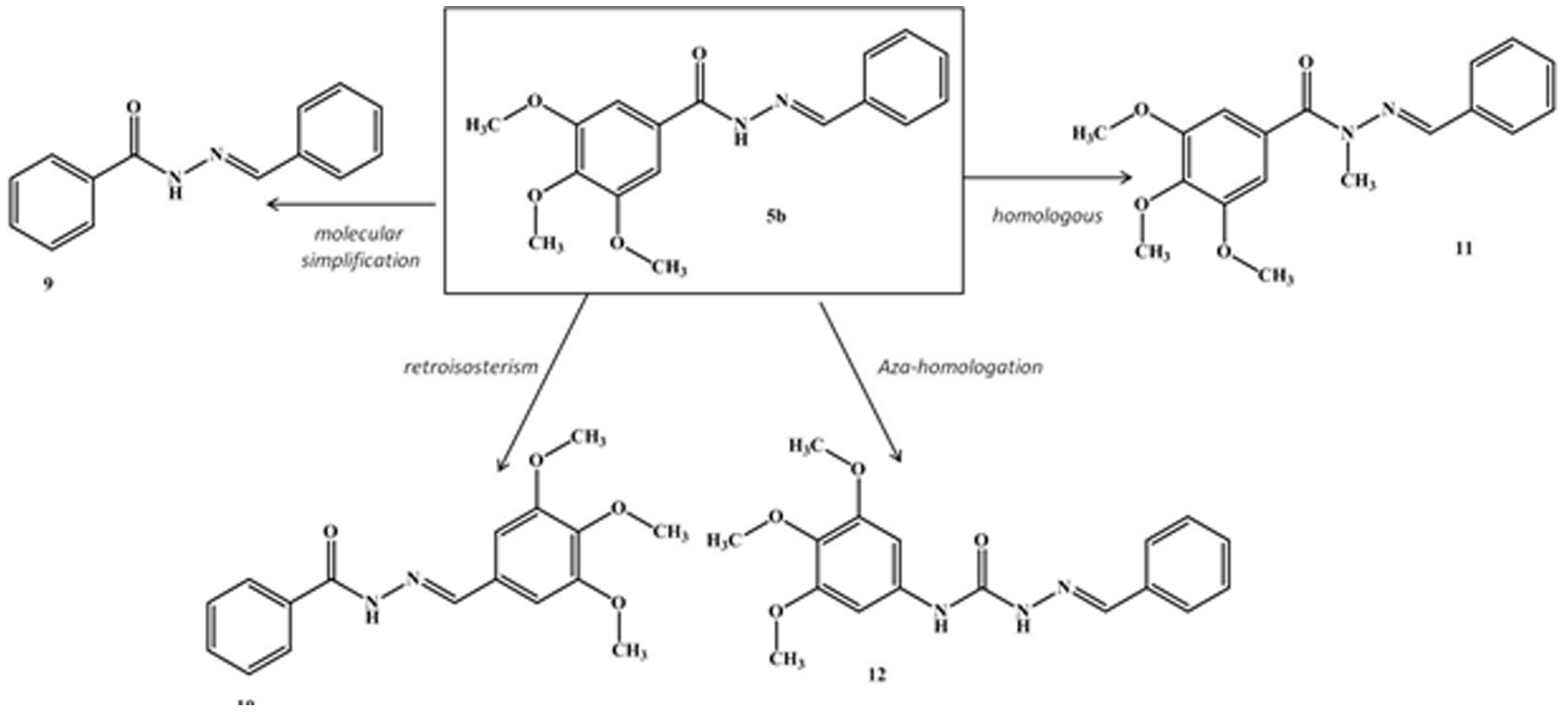
Design of compounds 9–12 from molecular modification of prototype 5b.

**Figure 7:**
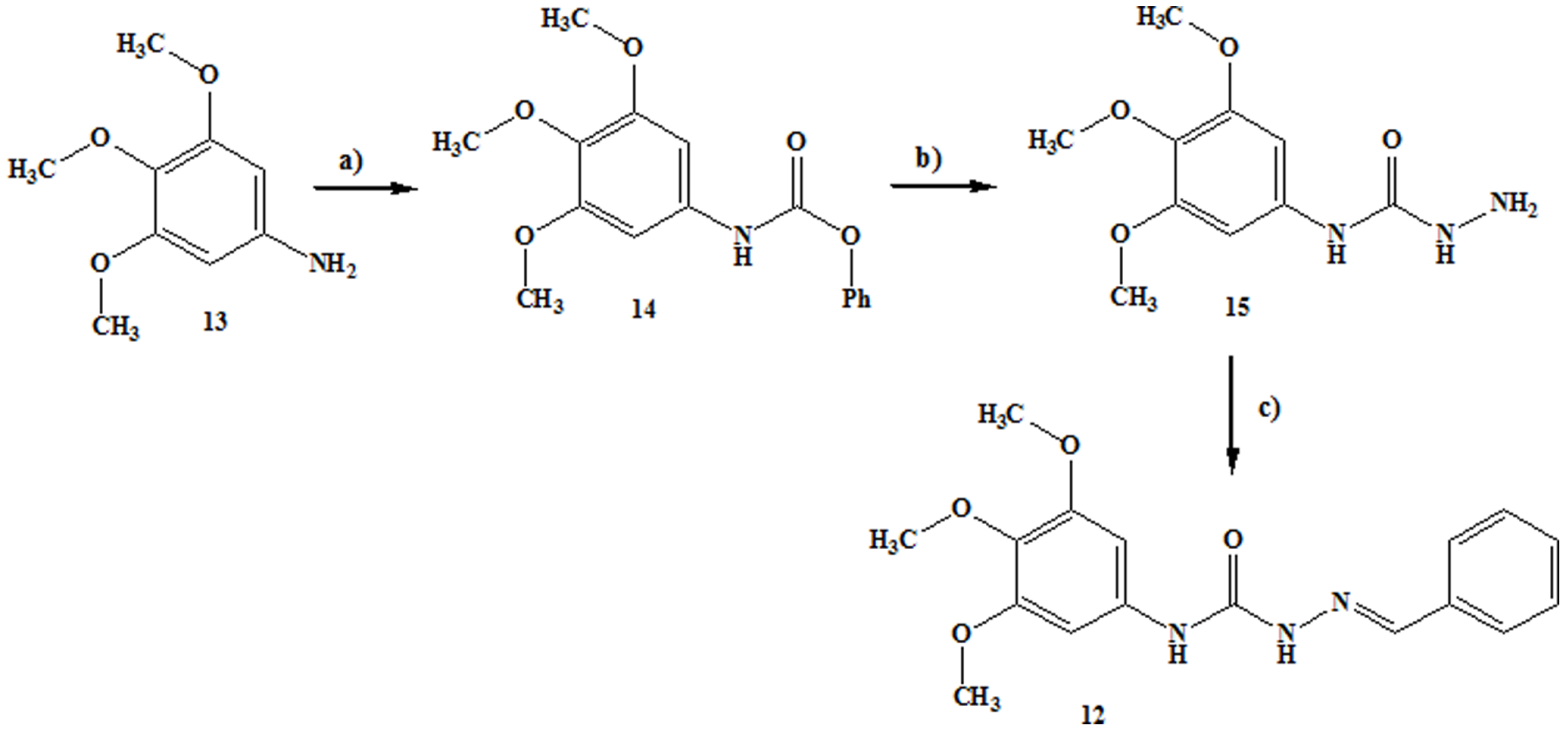
Conditions and reagents: a) Phenyl chloroformate, CHCl_3_, reflux, 2 h, 47%; b) N_2_H_4_.H_2_O, toluene, r.t., 72 h, 64%; c) PhCHO, EtOH, HCl (cat), r.t., 1 h, 83%.

To establish the minimum structural requirements essential for the anti-tubulin activity of LASSBio-1586 (**5b**), some molecular modifications were introduced to its structure, leading to the design of compounds **9–12** (Figure 6). The *N*-acylhydrazone derivatives **9** and **10** were synthesized using the same methodology employed to obtain compounds **5a–r** [19]. The homologous compound **11** was prepared in good yield via chemoselective alkylation of the sp^3^ nitrogen in the *N*-acylhydrazone functionality using methyl iodide and potassium carbonate in acetone [23]. Semicarbazone **12** was synthesized in three linear steps in 25% overall yield, as illustrated in Figure 7 [24].

**Figure 8:**
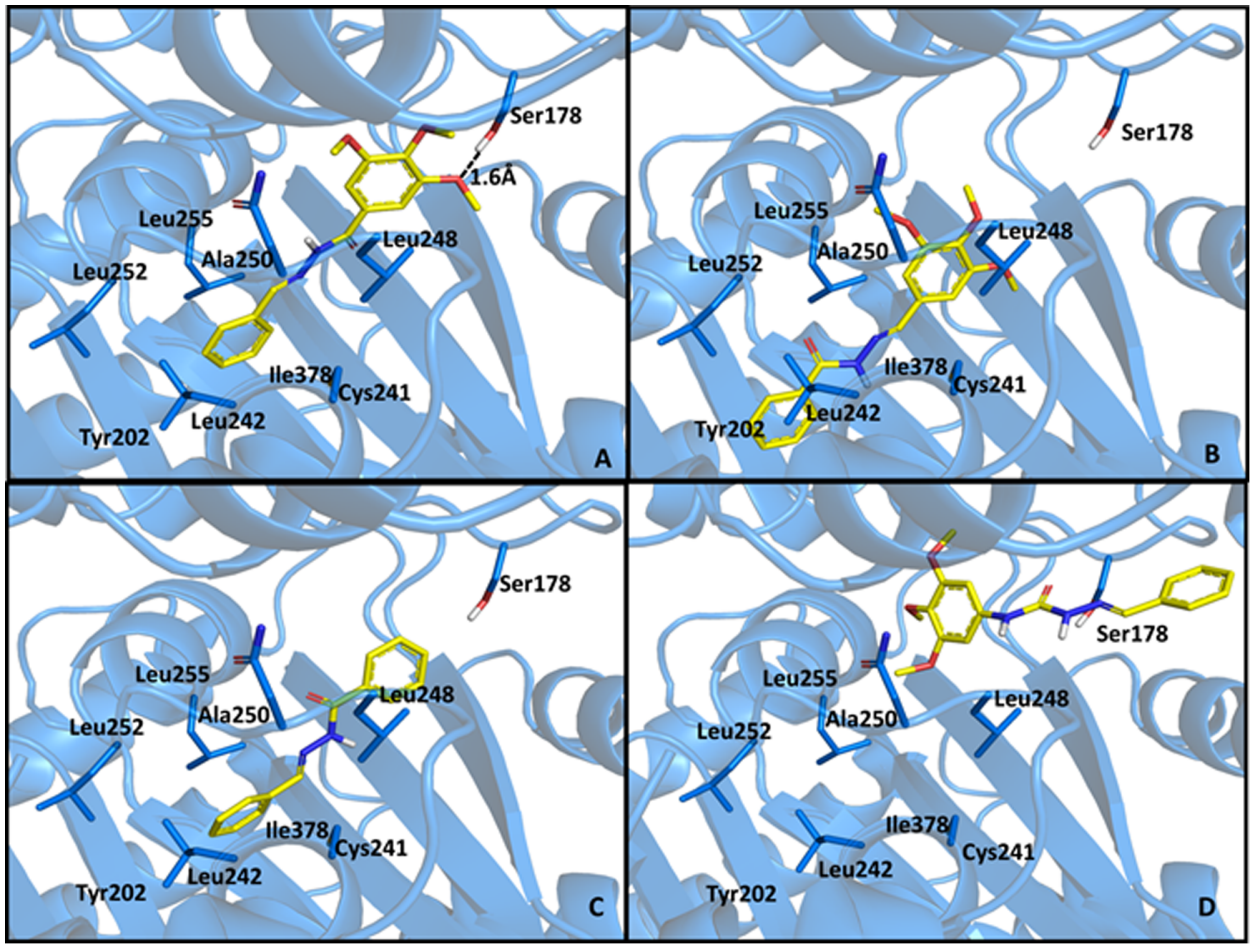
Best poses of compounds 5b (A), 10 (B), 9 (C) and 12 (D) at the colchicine binding pocket β-tubulin (PDB code: 1sa0).

The *in vitro* antiproliferative activity of compounds **9–12** was assessed against HL-60, SF296, HCT-8 and MDA-MB435 tumor cells and compared with the data from LASSBio-1586 (**5b**) and CA-4 (Table 4). As displayed in Table 4, the elimination of the methoxy groups from the trimethoxyphenyl subunit (ring A) present in LASSBio-1586 (**5b**) caused the loss of cytotoxic activity, as depicted by compound **9**, suggesting that this subunit was a pharmacophore. Similarly, retroisostere **10** was inactive, validating the role of the trimethoxyphenyl moiety as a pharmacophore when linked to the carbonyl group of the NAH functionality. The homologous compound **11** was well tolerated, exhibiting a slight increase in cytotoxic potency against HL-60 and HCT-8 tumor cell lines relative to compound **5b**. However, the aza-homologous **12** was inactive, suggesting that the semicarbazone unit was not suitable to replace the ethylene linker in CA4 or the NAH in **5b**. The greater conformational freedom introduced by the NH group may have compromised the bioactive conformation, altering the optimal spatial positioning between the aromatic rings necessary for molecular recognition with the β-tubulin binding site. To support these hypotheses, compounds **9–12** were subjected to docking studies and the best poses with the colchicine binding site of β-tubulin were analyzed (Figure 8). As presented in Figure 8, compounds **9** and **10** lost the hydrogen bond with Ser178 observed during the molecular interaction between compound **5b** and the colchicine binding pocket of β-tubulin protein. Similarly, semicarbazone **12** does not interact electrostatically with Ser178 and adopts a specific and unfavorable orientation within the active site of β-tubulin.

**Table 4:** Antiproliferative activity of compounds **CA-4**, **5b** and **9–12** against HL-60, SF295, HCT-8 and MDA-MB435 tumor cells.

IC_50_ (µM)				
Compound	HL-60	SF295	HCT-8	MDA-MB435
**CA-4**	0.0021	0.0062	0.0053	0.0079
**5b**	0.29	0.26	0.45	0.064
**9**	>25	>25	>25	>25
**10**	>25	>25	>25	>25
**11**	0.03	3.80	0.54	1.91
**12**	>25	>25	>25	>25

Considering the overall cytotoxic profile of LASSBio-1586 (**5b**) and its confirmed ability to inhibit microtubule polymerization, the antitumor activity was evaluated. The Hollow Fiber Assay (HFA) was selected because it is employed by the National Cancer Institute (NCI) as the standard model for the evaluation of new antiproliferative drugs before assessment via the *in vivo*-grown human tumor xenograft screen [25]–[28].

During the HFA, tumor cells (*i.e.*, SF-295 and HCT-116) were cultivated within biocompatible, semipermeable polyvinylidene fluoride hollow fibers (HFs) and subcutaneously (s.c.) implanted within the dorsal portion of BALB/c nude mice. LASSBio-1586 (**5b**) and 5-Fluorouracil (5-FU), which was the positive control, were administered intraperitoneally for 4 consecutive days. On day 5, the fibers were removed to quantify the antiproliferative activity of **5b** and 5-FU.

The hollow fibers were well tolerated by the animals, and no signs of rejection were detected. The treatments with LASSBio-1586 (**5b**) and 5-FU did not affect the health of the mice beyond acceptable limits and no deaths occurred.

As shown in Table 5, LASSBio-1586 (**5b**; dosages = 25 and 50 mg/kg/day) reduced the proliferation of both SF-295 (61.89 and 82.89%) and HCT-116 (72.68 and 80.76%) cell lines after 4 days of administration (P<0.05), demonstrating its antiproliferative effect *in vivo*.

**Table 5:** *In vivo* antiproliferative activity of **5b** and 5-fluorouracil (5-FU) in Hollow Fiber Assay (HFA).

Groups^1^	Dose (mg/kg/day)	Survival	Proliferation (OD595 nm)		Inhibition (%)	
			SF-295	HCT-116	SF-295	HCT-116
Control^2^	-	6/6	1.50±0.21	1.55±0.18	-	-
5-FU^3^	25	7/7	0.52±0.08*	0.59±0.10*	65.40	62.08
**5b**	25	7/7	0.57±0.05*	0.26±0.04*	61.89	82.89
	50	6/6	0.41±0.06*	0.29±0.05*	72.68	80.76

1 The data are reported as the mean ± S.E.M., n = 6–7 animals/group, which were treated for 4 days intraperitoneally.

2 The negative control group received 5% DMSO.

3 5-Fluorouracil (5-FU) was used as the positive control.

*P<0.05 compared to the control by ANOVA, followed by Newman-Keuls test.

## Conclusions

Based on the results of docking studies, a series of *N*-acylhydrazone derivatives were used as structural analogues of CA-4. These studies identified the major structural requirements essential for molecular recognition by the colchicine binding site of β-tubulin. Of the active compounds, LASSBio-1586 (**5b**) emerged as a simple antitumor drug candidate and was capable of inhibiting microtubule polymerization; this compound also possessed broad *in vitro* and *in vivo* antiproliferative profile and a better selectivity index than the lead compound, CA-4, which indicated that **5b** displayed improved selective cytotoxicity toward cancer cells.

## Methods

### Ethics Statement

Procedures are in accordance with guidelines for the welfare of animals in experimental neoplasia [40] and with national and international standard on the care and use of experimental laboratory animals [41] and were approved by the local Ethical Committee on Animal Research (Process No. 102/2007) at Federal University of Ceará (Fortaleza, Ceará, Brazil).

### Chemistry

Reagents and solvents were purchased from commercial suppliers and used as received. The reactions were monitored by thin layer chromatography, which was performed on aluminum sheets pre-coated with silica gel 60 (HF-254, Merck) to a thickness of 0.25 mm. The chromatograms were viewed under ultraviolet light (254–265 nm). For column chromatography Merck silica gel (70–230 mesh) was used. ^1^H NMR spectra were determined in deuterated dimethyl sulfoxide using a Bruker DPX-200 at 200 MHz. ^13^C NMR spectra were determined in this spectrometer at 50 MHz, employing the same solvent. Chemical shifts are given in parts per million (δ) from tretramethylsilane as internal standard, and coupling constant values (J) are given in Hertz (Hz). Signal multiplicities are represented by: s (singlet), d (doublet), t (triplet), q (quadruplet), m (multiplet) and br (broad signal). Infrared (IR) spectra were obtained with a FTLA 2000–100 spectrophotometer using potassium bromide plates.

Melting points of final products were determined with a Quimis 340 apparatus and are uncorrected. The purity of compounds were determined by HPLC (>95%) using the Shimadzu – LC20AD apparatus, a Kromasil 100-5C18 (4,6 mm×250 mm) column and the SPD-M20A detector (Diode Array) at 254 nm for quantification of analyte in a 1 mL/min constant flux. The injector was programmed to inject a volume of 20 -µL. The mobile phases used were: CH_3_CN∶H_2_O 1∶1; 6∶4 and 7∶3.

Ultraviolet spectroscopy was performed using Femto spectrophotometer. The wavelength used in solubility assay was determined by the -λ max characteristic of each compound. Spectra were analyzed in Femtoscan software. Mass spectrometry was obtained by positive ionization at Bruker AmaZon SL and data analyzed in Compass 1.3.SR2 software.

### General Procedure for the preparation of 3,4,5-trimethoxybenzohydrazide (8)

To a solution of methyl 3,4,5-trimethoxybenzoate (7) (2.00 g, 8.84 mmol) in absolute methanol (26 mL), 8.56 mL (176.8 mmol) of hydrazine hydrate 80% was added. The reaction mixture was kept under reflux for 5 hours, when TLC indicated the end of the reaction. Then, the media was poured into ice and the resulting precipitate was filtered out affording the 3,4,5-trimethoxybenzohydrazide in 88% yield, as a white solid, m.p. 166–168°C. The melting point, ^1^H NMR, ^13^C NMR and IR data are in agreement with previous reports [29]. I.R. (KBr) (cm^−1^): 3392, 3335, 3294, 3196 ν _sim._ and ν_assim._ NH), 1656 (ν CO), 1614 (ν NH); ^1^H NMR (200 MHz, DMSO-d_6_) δ (ppm): 9.72 (1 H, s, NH), 7.16 (2 H, s, H2 & H6), 4.47 (2 H, br, NH_2_), 3.81 (6 H, s, H3a & H5a), 3.69 (3 H, s, H4a); ^13^C NMR (50 MHz, DMSO-d_6_) δ (ppm): 165.4 (CO), 152.6 (C3 & C5), 139.8 (C4), 128.4 (C1), 104.5 (C2 & C6), 60.0 (C8), 55.9 (C7 & C9).

### General Procedure for the preparation of 3,4,5-trimethoxybenzoyl-arylhydrazones (5a–r)

To a solution of 8 (0.2 g, 0.884 mmol) in absolute ethanol (7 mL) containing one drop of 37% hydrochloric acid, was added 0.884 mmol of corresponding aldehyde derivative. The mixture was stirred at room temperature until TLC indicated the end of reaction (0.5–4 h). Then the mixture was poured into ice and the precipitate was filtered out and dried. Yields and characterization pattern are described below:

#### *(E)-N*′-(4-hydroxy-3-methoxybenzylidene)-3,4,5-trimetoxybenzohydrazide (5a; LASSBio-1593)

Yield: 72%, white solid, m.p. 117–120°C; I.R. (KBr) cm^−1^: 3220 (ν NH), 1635 (ν CO), 1579 (ν CN); ^1^H NMR (200 MHz, DMSO-d_6_) δ (ppm): 11.56 (1 H, s, NH), 9.35 (1 H, s, OH), 8.30 (1 H, s, N = CH), 7.28 (1 H, s, H2′), 7.22 (2H, s, H2 & H6), 7.08 – 6.95 (2 H, m, H5′ & H6′), 3.85 (6 H, s, H3a & H5a), 3.80 (3 H, s, H4a′), 3.72 (3 H, s, H4a); ^13^C NMR (50 MHz, DMSO-d_6_) δ (ppm): 162.4 (CO), 152.7 (C3 & C5), 149.9 (C4′), 148.0 (CN), 146.9 (C3′), 140.4 (C4), 128.7 (C1′), 127.2 (C1), 120.3 (C6′), 112.4 (C2′), 111.9 (C5′), 105.2 (C2 & C6), 60.2 (C4a), 56.1 (C3a & C5a), 55.6 (C4′a). 99.6% purity in HPLC (R.T. = 3.03 min; CH_3_CN∶H_2_O (7∶3)). MS: m/z = 361.1 (M+H)^+^.

#### *(E)-N*′benzylidene-3,4,5-trimetoxybenzohydrazide (5b; LASSBio-1586)

Yield: 76%, white solid, m.p. 131–134°C The melting point, ^1^H NMR, ^13^C NMR and IR data are in agreement with previous reports [29]. I.R. (KBr) (cm^−1^): 3183 (ν NH), 1648 (ν CO), 1584 (ν CN); ^1^H NMR (200 MHz, DMSO-d_6_) δ (ppm): 11.73 (1 H, s, NH), 8.48 (1 H, s, N = CH), 7.73 (2 H, d, J = 2 Hz, H2′ & H6′), 7.47- 7.45 (3 H, m, H3′,H4′ & H5′), 7.25 (2 H, s, H2 & H6), 3.87 (6 H, s, H3a & H5a), 3.73 (3 H, s, H4a); ^13^C NMR (50 MHz, DMSO-d_6_) δ (ppm): 162.6 (CO), 152.7 (C3 & C5), 147.8 (CN), 140.5 (C4), 134.3 (C1′), 130.0 (C4′), 128.8 (C2′ & C6′), 128.5 (C1), 127.0 (C3′ & C5′), 105.3 (C2 & C6), 60.1 (C4a), 56.1 (C3a & C5a). 99.4% purity in HPLC (R.T. = 3.89; CH_3_CN∶H_2_O (7∶3)). MS: m/z = 315.1 (M+H)^+^.

#### *(E)-N*′-(3-hydroxybenzylidene)-3,4,5-trimetoxybenzohydrazide (5c; LASSBio-1587)

Yield: 83%, cream solid, m.p. 227–229°C; I.R. (KBr) (cm^−1^): 3462 (ν OH), 3280 (ν NH), 1665 (ν CO), 1587 (ν CN); ^1^H NMR (200 MHz, DMSO-d_6_) δ (ppm): 11.68 (1 H, s, NH), 9.68 (1 H, s, OH), 8.37 (1 H, s, N = CH), 7.23 (4 H, m, H2, H6,), 7.11 (2 H, d, J = 10 Hz, H4′), 6.84 (2 H, d, J = 6 Hz, H6′), 3.86 (6 H, s, H3a & H5a), 3.73 (3 H, s, H4a); ^13^C NMR (50 MHz, DMSO-d_6_) δ (ppm): 162.6 (CO), 157.7 (C3′), 152.7 (C3 & C5), 147.9 (CN), 140.5 (C4), 135.6 (C1′), 129.9 (C5′), 128.5 (C1), 118.8 (C6′), 117.5 (C4′), 112.7 (C2′), 105.3 (C2 & C6), 60.2 (C4a), 56.1 (C5a & C3a). 97.5% purity in HPLC (R.T. = 3.12; CH_3_CN∶H_2_O (7∶3)). MS: m/z = 331.1 (M+H)^+^.

#### *(E)-N*′-(4-hydroxybenzylidene)-3,4,5-trimetoxybenzohydrazide (5d; LASSBio-1588)

Yield: 65%, pale yellow solid, m.p. 189°C; I.R. (KBr) cm^−1^: 3382 (ν OH), 3279 (ν NH), 1638 (ν CO), 1584 (ν CN); ^1^H NMR (200 MHz, DMSO-d_6_) δ (ppm): 11.51 (1 H, s, NH), 9.94 (1 H, s, OH), 8.36 (1 H, s, N = CH), 7.57 (2 H, d, J = 8 Hz, H3′ & H5′), 7.22 (1 H, s, H2 & H6), 6.84 (2H, d, J = 8 Hz, H2′ & H6′), 3.86 (6 H, s, H3a & H5a), 3.72 (3 H, s, H4a); ^13^C NMR (50 MHz, DMSO-d_6_) δ (ppm): 162.3 (CO), 159.4 (C4′), 152.7 (C3 & C5), 148.2 (CN), 140.3 (C4), 128.8 (C3′ & C5′), 128.7 (C1), 125.3 (C1′), 115.8 (C2′ & C6′), 105.2 (C2 & C6), 60.1 (C4a), 56.1 (C3a & C5a). 95.7% purity in HPLC (R.T. = 3.26 min; CH_3_CN∶H_2_O (6∶4)). MS: m/z = 331.1 (M+H)^+^.

#### *(E)-N*′-(3,4-dihydroxybenzylidene)-3,4,5-trimetoxybenzohydrazide (5e; LASSBio-1589)

Yield: 85%, white solid, m.p. 160°C; I.R. (KBr) cm^−1^: 3435 (ν OH), 3215 (ν NH), 1649 (ν CO), 1582 (ν CN)^;^^1^H NMR (200 MHz, DMSO-d_6_) δ (ppm): 11.48 (1 H, s, NH), 9.35 (2 H, sl, OH), 8.27 (s, 1 H, N = CH), 7.22 (3 H, m, H2, H6 & H2′), 6.95 (1H, d, J = 6 Hz, H5′), 6.89 (1H, d, J = 8 Hz, H6′), 3.86 (6 H, s, H3a & H5a), 3.72 (3 H, s, H4a); ^13^C NMR (50 MHz, DMSO-d_6_) δ (ppm): 162.2 (CO), 152.7 (C3 & C5), 148.3 (CN), 148.0 (C4′), 145.7 (C3′), 140.3 (C4), 128.7 (C1′), 125.7 (C1), 120.5 (C6′), 115.6 (C5′), 122.7 (C2′), 105.1 (C2 &C6), 60.1 (C4a), 56.1 (C3a & C5a). 99.0% purity in HPLC (R.T. = 2.88 min; CH_3_CN∶H_2_O (7∶3)). MS: m/z = 347.1 (M+H)^+^.

#### *(E)-N*′-(3-hydroxy-4-methoxybenzylidene)-3,4,5-trimetoxybenzohydrazide (5f; LASSBio-1592)

Yield: 62%, yellow solid, m.p. 210°C. The melting point, ^1^H NMR, ^13^C NMR and IR data are in agreement with previous reports [31]. I.R. (KBr) cm^−1^: 3223 (ν NH), 1638 (ν CO), 1582 (ν CN); ^1^H NMR (200 MHz, DMSO-d_6_) δ (ppm): 11.55 (1 H, s, NH), 9.56 (1 H, s, OH), 8.36 (1 H, s, N = CH), 7.32 (1 H, s, H2′), 7.23 (2 H, s, H2 & H6), 7.09 (1 H, d, J = 8 Hz, H5′), 6.85 (1H, d, J = 8 Hz, H6′), 3.86 (6 H, s, H3a & H5a), 3.83 (3 H, s, H3a′), 3.73 (3 H, s, H4a); ^13^C NMR (50 MHz, DMSO-d_6_) δ (ppm): 162.3 (CO), 152.6 (C3 &C5), 149.0 (C4′), 148.4 (CN), 148.0 (C3′), 140.3 (C4), 128.7 (C1′), 125.7 (C1), 122.1 (C6′), 115.4 (C5′), 109.0 (C2′), 105.1 (C2 & C6), 60.1 (C4a), 56.1 (C3a & C5a), 55.5 (C3′a). 97.3% purity in HPLC (R.T. = 3.32 min; CH_3_CN∶H_2_O (6∶4)). MS: m/z = 361.1 (M+H)^+^.

#### *(E)-N*′-(3,4-dimethoxybenzylidene)-3,4,5-trimetoxybenzohydrazide (5g; LASSBio-1590)

Yield: 86%, pale yellow solid, m.p. 190–191°C; I.R. (KBr) cm^−1^: 3221 (ν NH), 1647 (ν CO), 1582 (ν CN); ^1^H NMR (200 MHz, DMSO-d_6_) δ (ppm): 11.62 (1 H, s, NH), 8.40 (1 H, s, N = CH), 7.35 (1 H, s, H2′), 7.23 – 7.19 (3 H, m, H2, H6 & H5′), 7.03 (1H, d, J = 8 Hz, H6′), 3.86 (6 H, s, H3a & H5a), 3.81 (6 H, s, H3a′ & H4a′), 3.72 (3 H, s, H4a); ^13^C NMR (50 MHz, DMSO-d_6_) δ (ppm): 162.4 (CO), 152.7 (C3 & C5), 150.8 (C4′), 149.1 (C3′), 148.1 (CN), 140.5 (C4), 128.6 (C1′), 127.0 (C1), 121.8 (C6′), 111.5 (C2′), 108.3 (C4′), 105.2 (C2 & C6), 60.1 (C4a), 56.1(C3′a & C5′a), 55.5 (C4′a), 55.4 (C3′a). 97.6% purity in HPLC (R.T. = 3.78 min; CH_3_CN∶H_2_O (6∶4)). MS: m/z = 375.2 (M+H)^+^.

#### *(E)-N*′-(benzo[*d*][1], [3]dioxol-5-ylmethylene)-3,4,5-trimetoxybenzohydrazide (5h; LASSBio-1591)

Yield: 70%, white solid, m.p. 222–223°C. The melting point, ^1^H NMR, ^13^C NMR and IR data are in agreement with previous reports [30]. I.R. (KBr) cm^−1^: 3223 (ν NH), 1638 (ν CO), 1582 (ν CN); ^1^H NMR (200 MHz, DMSO-d_6_) δ (ppm): 11.63 (1 H, s, NH), 8.38 (1 H, s, N = CH), δ 7.31 (1 H, s, H4′), 7.23 – 7.16 (3 H, m, H2, H6 & H6′), 6.99 (1H, d, J = 8 Hz, H7′), 6.09 (2 H, s, O-CH_2_-O), 3.86 (6 H, s, H3a & H5a), 3.72 (3 H, s, H4a); ^13^C NMR (50 MHz, DMSO-d_6_) δ (ppm): 162.4 (CO), 152.7 (C3 & C5), 149.1 (C3′a), 148.0 (C7′a), 147.6 (CN), 140.4 (C4), 128.7 (C1′), 128.6 (C1), 123.2 (C6′), 108.2 (C4′), 104.6 (C2 & C6), 101.5 (C2′), 60.1 (C4a), 56.1 (C3a & C5a). 97.9% purity in HPLC (R.T. = 5.94 min; CH_3_CN∶H_2_O (1∶1)). MS: m/z = 359.1 (M+H)^+^.

#### *(E)*-3,4,5-trimethoxy-*N*′-(3,4,5-trimethoxybenzylidene)benzohydrazide (5i; LASSBio-1594)

Yield: 92%, pale yellow solid, m.p. 232°C The melting point, ^1^H NMR, ^13^C NMR and IR data are in agreement with previous reports [30]. I.R. (KBr) cm^−1^: 3210 (ν NH), 1641 (ν CO), 1579 (ν CN); ^1^H NMR (200 MHz, DMSO-d_6_) δ (ppm): 11.71 (1 H, s, NH), 8.42 (1 H, s, N = CH), 7.23 (2 H, s, H2 & H6), 7.03 (2 H, s, H2′ & H6′), 3.86/3.84 (12 H, 2s, H3a, H5a, H3a′ & H5a′), 3.73/3.71 (6 H, 2s, H4a & H4a′); ^13^C NMR (50 MHz, DMSO-d_6_) δ (ppm): 162.6 (CO), 153.2 (C3 & C5), 152.6 (C3′ & C5′), 147.9 (CN), 140.4 (C4), 139.1 (C4′), 129.8 (C1), 128.6 (C1′), 105.2 (C2 & C6), 104.3 (C2′ & C6′), 60.1 (C4a & C4′a), 56.1 (C3a & C5a), 55.9 (C3′a & C5′a). 96.0% purity in HPLC (R.T. = 3.41 min; CH_3_CN∶H_2_O (7∶3)). MS: m/z = 405.2 (M+H)^+^.

#### *(E)*-3,4,5-trimethoxy-*N*′-((4-oxo-4H-chromen-3-yl)methylene)benzohydrazide (5j; LASSBio-1595)

Yield: 95%, pale yellow solid, m.p. 205–206°C. I.R. (KBr) cm^−1^: 3222 (νNH), 1640 (ν CO), 1584 (ν CN); ^1^H NMR (200 MHz, DMSO-d_6_) δ (ppm): 11.80 (1 H, s, NH), 8.84 (1 H, s, N = CH), 8.65 (1 H, s, H2′), 8.13 (1 H, d, J = 8 Hz, H8′), 7.86 (1 H, t, J = 8 Hz, H6′), 7.72 (1 H, d, J = 8 Hz, H5′), 7.55 (1 H, t, J = 8 Hz, H7′), 7.26 (2 H, s, H2 & H6), 3.87 (6 H, s, H3a & H5a), 3.57 (3 H, s, H4a); ^13^C NMR (50 MHz, DMSO-d_6_) δ (ppm): 175.1 (C1′), 162.2 (CO), 155.7 (C3′), 154.5 (C4′a), 152.7 (C3 & C5), 140.5 (CN), 140.1 (C4), 134.6 (C6′), 128.1 (C1), 128.0 (C8′), 125.2 (C8′a), 123.3 (C7′), 118.7 (C5′), 118.3 (C2′), 105.2 (C2 & C6), 60.1 (C4a), 56.1 (C3a, C5a). 98.0% purity in HPLC (R.T. = 3.49 min; CH_3_CN∶H_2_O (7∶3)). MS: m/z = 383.1 (M+H)^+^.

#### *(E)-N*′-(3,5-di-tert-butyl-4hydroxybenzylidene)-3,4,5-trimetoxybenzohydrazide (5k; LASSBio-1596)

Yield: 67% after column chromatographic (dichloromethane: methanol), pale yellow solid, m.p. 222–224°C. I.R. (KBr) cm^−1^: 3207 (ν NH), 1646 (ν CO), 1583 (νCN); ^1^H NMR (200 MHz, DMSO-d_6_) δ (ppm): 11.49 (1 H, s, NH), 8.42 (1 H, s, N = CH), 7.48 (2 H, s, H2′ & H6′), 7.43 (1 H, s, OH), 7.23 (2 H, s, H2 & H6), 3.86 (6 H, s, H3a & H5a), 3.72 (3 H, s, H4a), 1.41 (18H, s, H5b′ & H3b′); ^13^C NMR (50 MHz, DMSO-d_6_) δ (ppm): 162.2 (CO),156.1 (C4′), 152.6 (C3 & C5), 149.4 (CN), 140.3 (C4), 139.4 (C3′ & C5′), 128.7 (C1), 125.5 (C1′), 123.87 (C2′ & C6′), 105.1 (C2 &C6), 60.1 (C4a), 56.0 (C3a & C5a), 34.4 (3a′), 30.1 (3b′). 98.9% purity in HPLC (R.T. = 7.56 min; CH_3_CN∶H_2_O (7∶3)). MS: m/z = 443.3 (M+H)^+^.

#### (*E*) -3,4,5-trimetoxy-*N*′-(naphtalen-1-ylmethylene) benzohydrazide (5l; LASSBio-1738)

Yield: 88%, white solid, m.p. >250°C The melting point, ^1^H NMR, ^13^C NMR and IR data are in agreement with previous reports [32]. I.R. (KBr) cm^−1^: 3226 (ν NH), 1646 (ν CO), 1591 (ν CN); ^1^H NMR (200 MHz, DMSO-d_6_) δ (ppm): 11.84 (1 H, s, NH), 9.12 (1 H, s, N = CH), 8.89 (1 H, d, J = 8 Hz, H2′), 8.04 – 7.93 (3 H, m, H4′, H5′ & H8′), 7.68 – 7.57 (3 H, m, H3′, H6′ & H7′), 7.31 (2 H, s, H2 & H6), 3.89 (6 H, s, H3a & H5a), 3.75 (3H, s, H4a); ^13^C NMR (50 MHz, DMSO-d_6_) δ (ppm): 162.6 (CO), 152.7 (C3 & C5), 147.4 (CN), 140.5 (C4), 133.5 (C4′a), 130.5 (C4′), 130.1 (C8′a), 129.6 (C1), 128.8 (C1′), 128.5 (C5′), 127.7 (C3′), 127.2 (C6′), 126.2 (C7′), 125.5 (C2′), 124.0 (C8′), 105.3 (C2 & C6), 60.1 (C4a), 56.1 (C3a, C5a). 97.0% purity in HPLC (R.T. = 4.95 min; CH_3_CN∶H_2_O (7∶3)). MS: m/z = 365.2 (M+H)^+^.

#### (*E*) -3,4,5-trimetoxy-*N*′-(naphtalen-2-ylmethylene) benzohydrazide (5m; LASSBio-1739)

Yield: 89%, white solid, m.p. >250°C. The melting point, ^1^H NMR, ^13^C NMR and IR data are in agreement with previous reports [32]. I.R. (KBr) cm^−1^: 3176 (ν NH), 1645 (ν CO), 1578 (ν CN); ^1^H NMR (200 MHz, DMSO-d_6_) δ (ppm): 11.84 (1 H, s, NH), 8.63 (1 H, s, N = CH), 8.15 (1 H, s, H1′), 8.04 (4 H, m, H3′, H4′, H5′ & H8′), 7.59 – 7.55 (2 H, m, H6′ & H7′), 3.88 (6 H, s, H3a & H5a), 3.74 (3H, s, H4a); ^13^C NMR (50 MHz, DMSO-d_6_) δ (ppm): 162.6 (CO), 152.6 (C3 & C5), 147.6 (CN), 133.7 (C4′a), 132.8 (C1′), 132.0 (C2′), 128.5 (C8′a), 128.5 (C8′ & C1), 128.3 (C6′), 127.7 (C4′), 127.1 (C5′), 126.7 (C7′), 122.6 (C3′), 105.2 (C2 & C6), 60.1 (C4a), 56.1 (C3a, C5a). 98.1% purity in HPLC (R.T. = 5.03 min; CH_3_CN∶H_2_O (7∶3)). MS: m/z = 365.2 (M+H)^+^.

#### (*E*)-*N*′-(biphenyl-4-ylmethylene)-3,4,5- trimethoxyibenzohydrazide (5n; LASSBio-1740)

Yield: 87%, white solid, m.p. 187–188°C. I.R. (KBr) cm^−1^: 3204 (ν NH), 1644 (ν CO), 1585 (ν CN); ^1^H NMR (200 MHz, DMSO-d_6_) δ (ppm): 11.77 (1 H, s, NH), 8.52 (1 H, s, N = CH), 7.86 – 7.71 (6 H, m, H3′, H5′, H2′, H6′, H6′a & H2′a), 7.52 – 7.39 (3 H, m, H3′a, H4′a & H5′a), 3.87 (H3a & H5a), 3.74 (H4a); ^13^C NMR (50 MHz, DMSO-d_6_) δ (ppm): 162.6 (CO), 152.7 (C3 & C5), 147.3 (CN), 141.6 (C1′), 140.5 (C4), 139.3 (C1′a), 133.4 (C4′), 129.0 (C3′ & C5′), 128.5 (C3′a & C5′a), 127.8 (C1), 127.6 (C2′ & C6′), 127.0 (C2′a & C6′a), 126.6 (C4′a), 105.3 (C2 & C6), 60.1 (C4a), 56.1 (C3a, C5a). 98.8 purity in HPLC (R.T. = 5.57 min; CH_3_CN∶H_2_O (7∶3)). MS: m/z = 391.2 (M+H)^+^.

#### (*E*)-3,4,5-trimethoxy-*N*′-(4-methylbenzylidene) benzohydrazide (5o; LASSBio-1741)

Yield: 83%, white solid, m.p. 189–190°C The melting point, ^1^H NMR, ^13^C NMR and IR data are in agreement with previous reports [32]. I.R. (KBr) cm^−1^: 3208 (ν NH), 1644 (ν CO), 1586 (ν CN); ^1^H NMR (200 MHz, DMSO-d_6_) δ (ppm): 11.66 (1 H, s, NH), 8.43 (1 H, s, N = CH), 7.63 (2 H, d, J = 8 Hz, H2′ & H6′), 7.29 – 7.24 (4 H, m, H3′, H5′, H2 & H6), 3.86 (6 H, s, H3a & H5a), 3.73 (3 H, s, H4a), 2.34 (3H, s, H4′a); ^13^C NMR (50 MHz, DMSO-d_6_) δ (ppm): 162.5 (CO), 152.7 (C3 & C5), 147.9 (CN), 140.5 (C4), 139.9 (C4′), 131.6 (C1′), 129.5 (C3′ & C5′), 128.6 (C1), 127.1 (C2′ & C6′), 105.2 (C2 & C6), 60.1 (C4a), 56.1 (C3a, C5a), 21.1 (C4′a). 96.7% purity in HPLC (R.T. = 4.12 min; CH_3_CN∶H_2_O (7∶3)). MS: m/z = 315.1 (M+H)^+^.

#### *(E)-N*′-(4-fluorobenzylidene) - 3,4,5-trimetoxybenzohyidrazide (5p; LASSBio-1742)

Yield: 82%, white solid, m.p.181–182°C The melting point, ^1^H NMR, ^13^C NMR and IR data are in agreement with previous reports [30]. I.R. (KBr) cm^−1^: 3185 (ν NH), 1649 (ν CO), 1588 (ν CN); ^1^H NMR (200 MHz, DMSO-d_6_) δ (ppm): 11.78 (1 H, s, NH), 8.47 (1 H, s, N = CH), 7.83 – 7.76 (2 H, m, H2′ & H6′), 7.35 – 7.24 (4 H, m, H3′, H5′, H2 & H6), 3.86 (6 H, s, H3a & H5a), 3.73 (3 H, s, H4a); ^13^C NMR (50 MHz, DMSO-d_6_) δ (ppm): 165.5 – 160.6 (C4′, J_CF_ = 246 Hz), 162.5 (CO), 152.7 (C3 & C5), 146.6 (CN), 140.5 (C4), 130.9 (C1′), 129.3 – 129.1 (C2′ & C6′, J_CF_ = 8,5 Hz), 128.4 (C1), 116.1 – 115.7 (C3′ & C5′, J_CF_ = 21,5 Hz), 105.2 (C2 & C6), 60.1 (C4a), 56.1 (C3a, C5a). 98.0% purity in HPLC (R.T. = 3.79 min; CH_3_CN∶H_2_O (7∶3)). MS: m/z = 333.1 (M+H)^+^.

#### *(E)-N*′-(4-chlorobenzylidene)-3,4,5-trimetoxybenzohyidrazide (5q; LASSBio-1743)

Yield: 83%, white solid, m.p.187–188°C. The melting point, ^1^H NMR, ^13^C NMR and IR data are in agreement with previous reports [32]. I.R. (KBr) cm^−1^: 3235 (ν NH), 1647 (ν CO), 1582 (ν CN), 1079 (ν Ar-Cl); ^1^H NMR (200 MHz, DMSO-d_6_) δ (ppm): 11.78 (1 H, s, NH), 8.46 (1 H, s, N = CH), 7.76 (2 H, d, J = 8 Hz, H2′ & H6′), 7.52 (2 H, d, J = 8 Hz, H3′& H5′), 7.24 (2 H, s, H2 & H6), 3.86 (6 H, s, H3a & H5a), 3.73 (3 H, s, H4a); ^13^C NMR (50 MHz, DMSO-d_6_) δ (ppm): 162.6 (CO), 152.7 (C3 & C5), 146.4 (CN), 140.5 (C4), 134.5 (C4′), 133.2 (C1′), 128.9 (C3′ & C5′), 128.6 (C2′& C6′), 128.3 (C1), 105.3 (C2 & C6), 60.1 (C4a), 56.1 (C3a, C5a). 97.5% purity in HPLC (R.T. = 4.36 min; CH_3_CN∶H_2_O (7∶3)). MS: m/z = 349.1 (M+H)^+^ and 351.1 ([M+2]+H)^+^.

#### *(E)-N*′-(4-bromobenzylidene) - 3,4,5-trimetoxybenzohyidrazide (5r; LASSBio-1744)

Yield: 78%, white solid, m.p.214–215°C. The melting point, ^1^H NMR, ^13^C NMR and IR data are in agreement with previous reports [30]. I.R. (KBr) cm^−1^: 3263 (ν NH), 1664 (ν CO), 1587 (ν CN), 1067 (ν Ar-Br); ^1^H NMR (200 MHz, DMSO-d_6_) δ (ppm): 11.78 (1 H, s, NH), 8.44 (1 H, s, N = CH), 7.67 (4 H, s, H2′, H3′, H5′ & H6′), 7.24 (2 H, s, H2 & H6), 3.86 (6 H, s, H3a & H5a), 3.73 (3 H, s, H4a); ^13^C NMR (50 MHz, DMSO-d_6_) δ (ppm): 162.5 (CO), 152.6 (C3 & C5), 146.4 (CN), 140.5 (C4), 133.6 (C1′), 131.8 (C3′ & C5′), 128.8 (C2′ & C6′), 128.3 (C1), 123.2 (C4′), 105.3 (C2 & C6), 60.1 (C4a), 56.1 (C3a, C5a).98.5% purity in HPLC (R.T. = 4.74; CH_3_CN∶H_2_O (7∶3)). MS: m/z = 393.1 (M+H)^+^ and 395.1 ([M+2]+H)^+^.

### Benzohydrazide

A solution of benzoic acid (2.0 g, 16.4 mmol) in 50 mL of methanol, containing 5 drops of sulfuric acid, were refluxed with a Dean-Stark apparatus for 5 hours to obtain the corresponding methyl ester. Then, 328 mmol of hydrazine hydrate 80% were added to the reaction mixture and kept under reflux for 2 hours to obtain benzohydrazide in a one-pot methodology in 94% yield as a white solid, m.p. 112–114°C. The melting point data is in agreement with previous reports [33].

#### (*E*)-*N*′-benzylidenebenzohydrazide (9; LASSBio-372)

Yield: 53%, cream solid, m.p. 211–212°C. The melting point, ^1^H NMR, ^13^C NMR and IR data are in agreement with previous reports [34]. I.R. (KBr) cm^−1^: 3181 (ν NH), 1641 (ννCO), 1600 (ν CN); ^1^H NMR (200 MHz, DMSO-d_6_) δ (ppm): 11.86 (1 H, s, NH), 8.48 (1 H, s, N = CH), 7.93 (2 H, d, J = 6 Hz, H2 & H6), 7.73 (2 H, d, J = 4 Hz, H2′ & H6′), 7.60 – 7.45 (6 H, m, H3, H4, H5, H5′, H4′ & H3′); ^13^C NMR (50 MHz, DMSO-d_6_) δ (ppm): 163.1 (CO), 147.7 (CN), 134.3 (C1), 133.4 (C1′), 131.6 (C4), 130.0 (C4′), 128.8 (C2 & C6), 128.4 (C2′ & C6′), 127.5 (C3 & C5), 127.0 (C3′ & C5′); 97.8% purity in HPLC (R.T. = 3.78; CH_3_CN∶H_2_O (7∶3)). MS: m/z = 225.1 (M+H)^+^.

#### *(E)-N*′-(3,4,5-trimethoxybenzylidene)-benzohydrazide (10; LASSBio-1734)

Yield: 56%, cream solid, m.p. 211–212°C; I.R. (KBr) cm^−1^: 3239 (ν NH), 1649 (ν CO), 1575 (ν CN); RMN ^1^H (200 MHz, DMSO-d_6_) δ (ppm): 11.86 (1 H, s, NH), 8.39 (1 H, s, N = CH), 7.91 (2 H, d, J = 8 Hz, H2 & H6), 7.62 -7.51 (3 H, m, H3, H4 & H5), 7.03 (2 H, s, H2′ & H6′), 3.84 (6 H, s, C3′a & C5′a), 3.35 (C4′a); ^13^C NMR (50 MHz, DMSO-d_6_) δ (ppm): 163.1 (CO), 153.1 (C3′ & C5′), 147.8 (CN), 139.2 (C4′), 133.5 (C1), 131.6 (C1′), 129.8 (C4), 128.4 (C2 & C6), 127.6 (C3 & C5), 104.3 (C2′ & C6′), 60.1 (C4′a), 55.9 (C3′a & C5′a); 98.3% purity in HPLC (R.T. = 3.47; CH_3_CN∶H_2_O (7∶3)). MS: m/z = 315.1 (M+H)^+^.

#### *(E)- N*′-benzylidene-3,4,5-trimethoxy-*N*-methylbenzohydrazide (11; LASSBio-1735)

To a solution of LASSBio-1586 (0.4 g, 1.27 mmol) in 7 mL of acetone was added 3.82 mmol of sodium carbonate. The resultant suspension was stirred at room temperature for 50 minutes. Then, methyl iodide (0.48 mL, 7.63 mmol) was added to the suspension and the reaction mixture was heated for 24 hours at 40°C. After total conversion of reactant to product, the acetone were removed under reduced pressure and then the material were suspended in 2 mL of ethanol, filtered and washed with petroleum ether. Recrystallization of the *N*-methylated product was performed in ethanol/water mixture. LASSBio-1735 was obtained in 94% of yield as a white crystalline solid with cotton aspect. m.p.71–73°C; I.R. (KBr) cm^−1^: 1648 (ν CO), 1592 (ν CN); ^1^H NMR (200 MHz, DMSO-d_6_) δ (ppm): 8.04 (1 H, s, N = CH), 7.58 (2 H, d, J = 8 Hz, H2′ & H6′), 7.41 – 7.38 (3 H, m, H3′, H4′ & H5′), 7.00 (2 H, s, H2 & H6), 3.77 (6 H, s, H3a & H5a), 3.75 (3 H, s, H4a), 3.50 (3 H, s, NCH_3_); ^13^C NMR (50 MHz, DMSO-d_6_) δ (ppm): 169.1 (CO), 151.8 (C3 & C5), 140.4 (CN), 139.2 (C4), 134.9 (C1′), 130.3 (C4′), 129.5 (C1), 128.7 (C2′ & C6′), 126.8 (C3′ & C5′), 107.6 (C2 & C6), 60.1 (C4a), 55.9 (C3a & C5a); 97.8% purity in HPLC (R.T. = 5.53; CH_3_CN∶H_2_O (7∶3)). MS: m/z = 329.1 (M+H)^+^.

### Synthesis of phenyl 3,4,5-trimetoxyphenylcarbamate (14)

3,4,5-trimethoxy aniline, **13**, (2.0 g, 10.92 mmol) dissolved in 20 mL of chloroform were add drop wised to a solution of phenylchloroformate (1.4 mL, 10.92 mmol) in 20 mL of chloroform. The resultant suspension was refluxed until total conversion of aniline to the corresponding carbamate. When at room temperature, 15 mL of *n*-hexane were added and the suspension were filtered under vacuum and washed with *n*-hexane. The compound **14** was obtained in 48% yield as cream needles, m.p. 170–171°C. The melting point, ^1^H NMR, ^13^C NMR and IR data are in agreement with previous reports [35]. I.R. (KBr) cm^−1^: 3334 (ν NH), 1717 (ν CO); ^1^H NMR (200 MHz, DMSO-d_6_) δ (ppm): 10.12 (1 H, s, Ar-NH), 7.46 – 7.39 (2 H, m, H3′ & H5′), 7.29 – 7.18 (3 H, m, H2′, H4′ & H6′), 6.88 (2 H, s, H2 & H6), 3.73 (6 H, s, H3a & H5a), 3.62 (3 H, s, H4a); ^13^C NMR (50 MHz, DMSO-d_6_) δ (ppm): 152.9 (C3 & C5), 151.8 (C1′), 150.5 (CO), 134.7 (C4), 133.4 (C1), 129.4 (C3′ & C5′), 125.5 (C4′), 122.0 (C2′ & C6′), 96.5 (C2 & C6), 60.1 (C4a), 55.8 (C3a & C5a).

### Synthesis of *N*-(3,4,5-trimetoxyphenyl) hydrazine carboxamide (15)

To a suspension of 14 (0.6 g, 1.98 mmol) in 15 mL of dry toluene, was added 29.7 mmol of hydrazide hydrate 64% and the mixture was stirred at room temperature until conversion of carbamate to correspondent semicarbazide. The product was filtered under vacuum, washed with *n*-hexane and obtained as a brown solid in 90% yield, m.p. >250°C; I.R.(KBr) cm^−1^: 3582, 3459, 3346, 3145 (ν NH), 1718 (ν CO-ester), 1684 (ν CO amide); ^1^H NMR (200 MHz, DMSO-d_6_) δ (ppm): 8.83 (1 H, s, NH), 7.75 (1 H, Ar-NH), 6.89 (2 H, s, H2 & H6), 3.71 (11 H, br, NH_2_, H3a, H4a, H5a); ^13^C NMR (50 MHz, DMSO-d_6_) δ (ppm): 156.9 (CO), 152.8 (C3 & C5), 135.9 (C4), 132.5 (C1), 96.2 (C2 & C6), 60.2 (C4a), 55.8 (C3a & C5a).

#### *(E)*-2-benzylidene-*N*-(3,4,5-trimetoxyphenyl) hydrazinecarboxamide (12; LASSBio-1714)

To a solution of 15 (0.2 g, 0.83 mmol) in etanol (7 mL), containing one drop of 37% chloridric acid, was added 0.83 mmol of benzaldehyde. The mixture was stirred at room temperature until TLC indicates the end of reaction. The mixture was poured into ice and the precipitate was filtered out and dried. LASSBio-1714 was obtained as a white solid in 83% yield, m.p. 217°C; I.R. (KBr) cm^−1^: 3371, 3193 (ν NH), 1685 (ν CO); ^1^H NMR (200 MHz, DMSO-d_6_): δ 10.73 (1 H, s, NH), 8.79 (1 H, s, Ar-NH), 7.97 (1 H, s, N = CH), 7.85 (2 H, d, J = 6 Hz, H2′ & H6′), 7.44 – 7.41 (3 H, m, H3′, H4′ & H5′), 7.11 (2 H, s, H2 & H6), 3.76 (6 H, s, H3a & H5a), 3.62 (3H, s, H4a); ^13^C NMR (50 MHz, DMSO-d_6_): δ 152.9 (CO), 152.6 (C3 & C5), 140.9 (CN), 135.2 (C4), 134.2 (C4′), 132.9 (C1′), 129.4 (C1), 128.6 (C2′ & C6′), 127.0 (C3′ & C5′) 97.7 (C2 & C6), 60.1 (C4a), 55.8 (C3a & C5a); 99.0% purity in HPLC (R.T. = 4.23 min; CH_3_CN∶H_2_O (7∶3)). MS: m/z = 330.1 (M+H)^+^.

### X-ray Crystallography

A colorless prismatic single crystal of the compound LASSBio-1586, suitable for x-ray study, was obtained by slow evaporation of a solution of methanol-dimethylformamide (2∶1) at room temperature 295(2) K. Data collection was performed using the Kappa Apex II Duo diffractometer operating with Cu-Kα radiation at 100 K. 8336 data points were collected of what 2687 are symmetry independent (R_int_ = 0.044). The molecule crystallizes in the *Pca2_1_* space group, having Z = 4. Structure solution was obtained using Direct Methods implemented in SHELXS [36] and the model refinement was performed with full matrix least squares on F^2^ using SHELXL [36], with final residuals R1 = 0.037, wR2 = 0.105 for 2461 observed data with I>2σ(I), and R1 = 0.046, wR2 = 0.111 for all data. The data completeness allowed for a qualitative decision of the chirality, however because only low weight atoms are present, the Flack parameter has a relatively large standard deviation, being 0.04(19). The crystal packing is stabilized by an intermolecular hydrogen bond of type N1–H1…O1^i^, building a linear chain though (100). Hydrogen bond geometry is given in Table 6. The programs ORTEP-3 [20], SHELXS/SHELXL [36] were used within WinGX ^37^ software package.

**Table 6:** Intermolecular hydrogen bond geometry.

D—H…A	D—H (Å)	H…A (Å)	D…A (Å)	D—H…A (°)	Symmetry operation
N1-H1…O1^i^	0.84(3)	2.12(3)	2.949(2)	170(2)	i) ½+x, 1-y, z

### Crystallographic data Information

Crystallographic data of compound **5b** (excluding structure factors) have been deposited with the Cambridge Crystallographic Data Centre as supplementary publication number CCDC 940524. Copies of the data can be obtained, free of charge, on application to CCDC, 12 Union Road, Cambridge CB2 1EZ, UK [fax: C44 1223 336033 or e-mail: deposit@ccdc.cam.ac.uk].

### Solubility Assay

The solubility assay was performed considering the absorptivity of compounds in ultraviolet spectroscopy as described by Schneider and coworkers [38]. The assay wavelength was determined by the λ max characteristic of each compound.

Saturated aqueous solutions were prepared (0.01 mg/mL) and were kept under stirring for 2 hours at 37°C. The supernatant was filtered in 0,45 µm filters and transferred to a quartz cuvette (10 mm) to spectra acquisition.

Solubility was determined by linear regression using as graph plots, solutions prepared by dilutions of the original solution in methanol. The data were obtained in triplicates and the mean values were used to the graph plots. The correlation coefficient (R^2^) values were between 0.9972 and 0.9999.

### *In Vitro* Antiproliferative Assay

Compounds (0.009–5 µg/mL) were tested for cytotoxic activity against selected cancer cell lines: SF-295 (glioblastoma), HCT-8 (colon), MDAMB-435 (melanoma), HL60 (leukemia), PC3M (prostate cancer), OVCAR-8 (ovaries adenocarcinoma) and NCI-H258M (pulmonary bronchio-alveolar carcinoma). All cell lines were kindly obtained from the National Cancer Institute (Bethesda, MD, USA).Tumor cell proliferation was quantified through the ability of living cells to reduce the yellow dye 3-(4,5-dimethyl-2-thiazolyl)-2,5-diphenyl-*2H*-tetrazolium bromide (MTT, Sigma Aldrich)) to a purple formazan product and absorbance was measured at 595 nm (DTX-880, Beckman Coulter) [21].

### Tubulin Polymerization Assay

The tubulin polymerization assay was performed by CEREP®, as described by Bonne and co-workers [39].

### Hollow Fiber Assay

A total of 26 female BALB/c nude (nu/nu) mice aging 6–8 weeks were obtained from the animal facilities of State University of São Paulo (USP), Faculty of Medicine, São Paulo (SP), Brazil. They were kept in well-ventilated and sterile cages (Alesco, São Paulo) under standard conditions of light (12 h with alternative day and night cycles) and temperature (22±1°C) and were housed with access to commercial sterile rodent stock diet (Nutrilabor, São Paulo, Brazil) and water *ad libitum*. As previously mentioned procedures are in accordance with guidelines for the welfare of animals in experimental neoplasia [40] and with national and international standard on the care and use of experimental laboratory animals [41] and were approved by the local Ethical Committee on Animal Research (Process No. 102/2007).

### Cell Culture

Cell culture of SF-295 (glioblastoma) and HCT-116 (colon carcinoma) was performed in RPMI 1640 medium supplemented with 10% fetal bovine serum, 2 mM glutamine, at 37°C with 5% CO_2_.

### HF Preparation, Surgery Deployment and Determination of the Antiproliferative Capacity

Polyvinylidene fluoride (PVDF) HFs with a 1-mm internal diameter and a molecular weight cutoff point of 500 kDa were used (Spectrum Laboratories, Houston, TX). The fibers were cut into pieces 12–15 cm long, washed 2× with sterile distilled water and kept in sterile conditions.

Before use, under sterile conditions, the fibers were incubated in complete RPMI with 20% fetal bovine serum (FBS) overnight (packaging time). Cell viability was assessed by trypan blue exclusion assay. Then, a cell suspension of 7.0×10^6^ cell/mL at 4°C was injected into the fiber, with the ends thereof immediately heat-sealed. The fibers were cut into 2 cm each, transferred to petri plates and incubated in complete RPMI medium for 24 h prior to implantation in mice. Each cell was injected into one fiber of a different color (HCT-116, yellow fibers; SF-295, blue fibers).

Mice were anaesthetized with ketamine (90 mg/kg) - xylazine (4.5 mg/kg) (Sigma Aldrich). Groups were divided into: a) Negative control (DMSO 5%, n = 6); b) Positive control (5-Flouoruracil, 5-FU, 25 mg/kg/day, n = 7) (Sigma Aldrich); c) LASSBio-1586 (**5b**; 25 mg/kg/day, n = 7); d) LASSBio-1586 (**5b**; 50 mg/kg/day, n = 6). A small incision in the neck was incised to permit subcutaneous (s.c.) implantation of the fibers in the dorsal part of the animal. Each animal received 2 fibers at s.c. site. All incisions were sealed with a surgical stapler. The test compounds were administered intraperitoneally during 4 consecutive days. On day 5, fibers were removed to quantify the antiproliferative capacity as described above.

### *In Vivo* Antiproliferative Assay

Tumor cell proliferation was quantified through the ability of living cells to reduce the yellow dye 3-(4,5-dimethyl-2-thiazolyl)-2,5-diphenyl-*2H*-tetrazolium bromide (MTT, Sigma Aldrich) to a purple formazan product [21]. For this purpose, the fibers removed from animals were incubated with MTT 1 mg/mL in 6-well plates during 4 h at 37°C, 5% CO_2_ and 95% humidity. The MTT solution was aspirated; fibers were washed with saline solution containing protamine sulphate 2.5% and incubated in protamine solution overnight at 4°C. Fibers were transferred to 24 well plates, cut into 2 or 3 pieces and put to dry. The formazan was dissolved in 500 mL of DMSO, aliquots (150 uL) were transferred to 96 well plates and absorbance was measured at 595 nm (DTX-880, Beckman Coulter).

### Statistical Analysis

In order to determine differences between groups, data (mean ± S.E.M) were compared by one-way analysis of variance (ANOVA) followed by Student Newman-Keuls test (P<0.05).

### Molecular Modeling

Compounds were constructed and submitted to a conformational analysis by molecular mechanics (MMFF method) with the Spartan 8.0 software (Wavefunction Inc.; Licence number: DQAIR/HASPUSB). The most stable conformer of each structure was reoptimized with the AM1 semiempirical molecular orbital method [42] and saved as mol2 files for docking studies into the colchicine binding site of the β-tubulin crystallographic structure available in the Protein Data Bank with code 1sa0. This structure was chosen because of the β-tubulin conformation induced by the co-crystallized colchicine, which prevents curved β-tubulin from adopting a straight structure, inhibiting assembly. Docking studies were implemented with the GOLD 5.0.1 program (CCDC), which employs a genetic algorithm for docking flexible ligands into protein binding sites and ranks the resulting poses according to their scores determined by available scoring functions. Hydrogen atoms were added to the protein structure according to the tautomeric and ionized states inferred by the program. The colchicine structure was removed for the docking studies, which were performed with the ChemScore scoring function, which contains specific energy terms for hydrogen bonding and lipophilic interactions [43], [44]. The data and poses were analyzed on Pymol program. Licences numbers: Pymol (8588); Gold (G/414/2006).

## Supporting Information

Figure S1 **β-tubulin polymerization assay performed by CEREP.**
(TIF)

Figure S2 **The pose of CA-4 Z-isomer (A) and E-isomer (B) at colchicine binding site of β-tubulin protein (PDB:1sa0).**
(TIF)

Figure S3 **Scatter plots (score x cLogP and score x molecular weight).**
(TIF)

Figure S4 **Compounds 5i (A), 5k (B), 5n (C) and 11 (D) poses at colchicine binding site of β-tubulin protein (PDB:1sa0).**
(TIF)
