# Supplementary figures and images for: Docking, Synthesis and Antiproliferative Activity of N-Acylhydrazone Derivatives Designed as Combretastatin A4 Analogues
Source: PLoS One. 2014 Mar 10;9(3):e85380. doi: 10.1371/journal.pone.0085380 (PMC3948622; doi:10.1371/journal.pone.0085380)

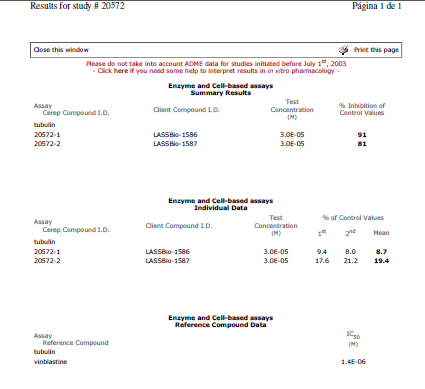

Supplement: Figure S1 — β-tubulin polymerization assay performed by CEREP. (TIF) [file pone.0085380.s001.tif]

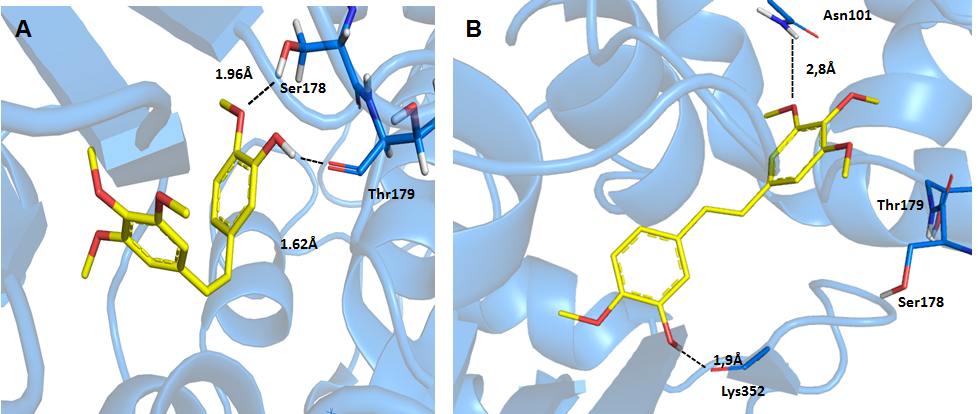

Supplement: Figure S2 — The pose of CA-4 Z-isomer (A) and E-isomer (B) at colchicine binding site of β-tubulin protein (PDB:1sa0). (TIF) [file pone.0085380.s002.tif]

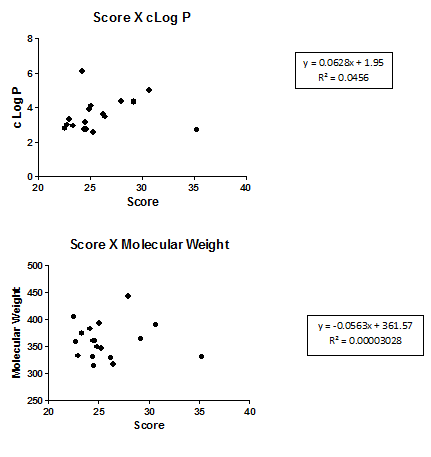

Supplement: Figure S3 — Scatter plots (score x cLogP and score x molecular weight). (TIF) [file pone.0085380.s003.tif]

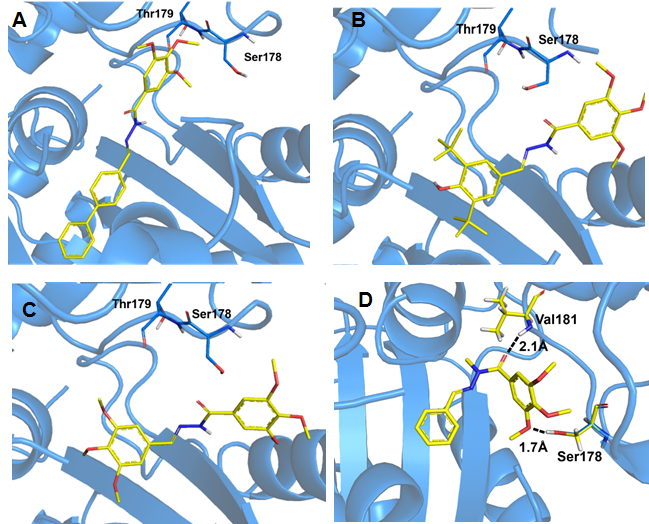

Supplement: Figure S4 — Compounds 5i (A), 5k (B), 5n (C) and 11 (D) poses at colchicine binding site of β-tubulin protein (PDB:1sa0). (TIF) [file pone.0085380.s004.tif]
